# Quantum-Scale Friction at Solid–Liquid Interface: Simulation, Detection Techniques, Mechanisms, and Emerging Applications

**DOI:** 10.1007/s40820-026-02066-2

**Published:** 2026-02-03

**Authors:** Yishu Han, Rui Zhang, Dameng Liu, Jianbin Luo

**Affiliations:** State Key Laboratory of Tribology in Advanced Equipment, Tsinghua University, Beijing, 100084 People’s Republic of China

**Keywords:** Solid–liquid interface, Quantum-scale friction, Interfacial drag reduction, Nanofluidic system

## Abstract

Solid–liquid interfaces are ubiquitous in nature and engineering, and their frictional behavior remains a key factor limiting performance gains in surface engineering. However, conventional tribology has largely focused on the effect of macroscopic variables such as surface topography, which do not account for the microscopic essence of ultra-low-friction phenomena at the nanoscale. Recently, the role of quantum-scale excitations, such as electrons and phonons, in micro-/nanoscale solid–liquid friction has been increasingly emphasized. By using in situ detection techniques such as terahertz time-domain spectroscopy and non-contact atomic force microscopy, the quantum-scale friction has been observed. Its essence stems from the energy and momentum transfer induced by fluctuations in liquid charge density or electron or phonon excitations within solids. However, limited capabilities in simultaneously probing multiple physical quantities at sub-nanometer and femtosecond resolutions hinder a comprehensive understanding of the quantum origins and applications of solid–liquid interfacial friction. This review synthesizes the cutting-edge theories and experimental advances in quantum-scale solid–liquid friction and proposes a potential breakthrough path based on deep integration of simulation and experiment to address core gaps, including incomplete theoretical frameworks and constrained detection capabilities. Despite multidimensional challenges, quantum-scale friction research demonstrates substantial potential for transformative technologies, such as low-power nanofluidic devices, high-efficiency energy storage, intelligent drug delivery, and super-lubrication materials, underscoring its significance for the convergence of interfacial science, quantum mechanics, and micro/nanofluidics.

## Highlights

- Reveals the quantum origin of solid liquid friction, governed by electron transfer, electron excitation, and electron-phonon coupling at interfaces.
- Summarizes emerging characterization techniques and multiscale simulations that uncover quantum scale friction mechanisms beyond classical tribology.
- Demonstrates the potential transformative applications of quantum scale interfacial friction in nano fluidics, energy harvesting, smart biomedical systems, and super lubrication.

## Graphical abstract

**Figure:**
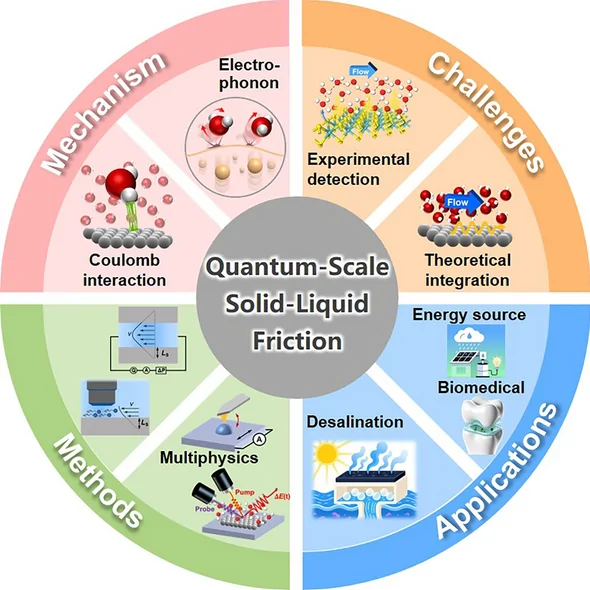

## Introduction

Solid–liquid interfaces are widespread in key areas such as energy engineering, marine vessels, lubricated power-transmission systems, and fluid transport systems, and have been extensively studied within fluid mechanics and interfacial science. However, drag at these interfaces leads to substantial energy losses, thereby affecting the operational efficiency of the systems. For example, maritime transport accounts for ~ 12% of global transport energy consumption, and approximately 60%–80% of energy losses within this sector are attributable to solid–liquid boundary-layer friction [1, 2]. Consequently, reducing interfacial drag and improving energy efficiency have been common objectives globally. Studies have demonstrated that interfacial slip can markedly reduce frictional drag and substantially increase energy utilization efficiency. For example, a slip length of 50 nm has been reported to raise interfacial energy conversion efficiency from 3% to 70% [3–5]. Accordingly, elucidating slip and friction mechanisms at solid–liquid interfaces and developing precise control strategies constitute core scientific and technological imperatives for overcoming bottlenecks in high-efficiency fluid transport systems and next-generation nanodevices.

**Fig. 1:**
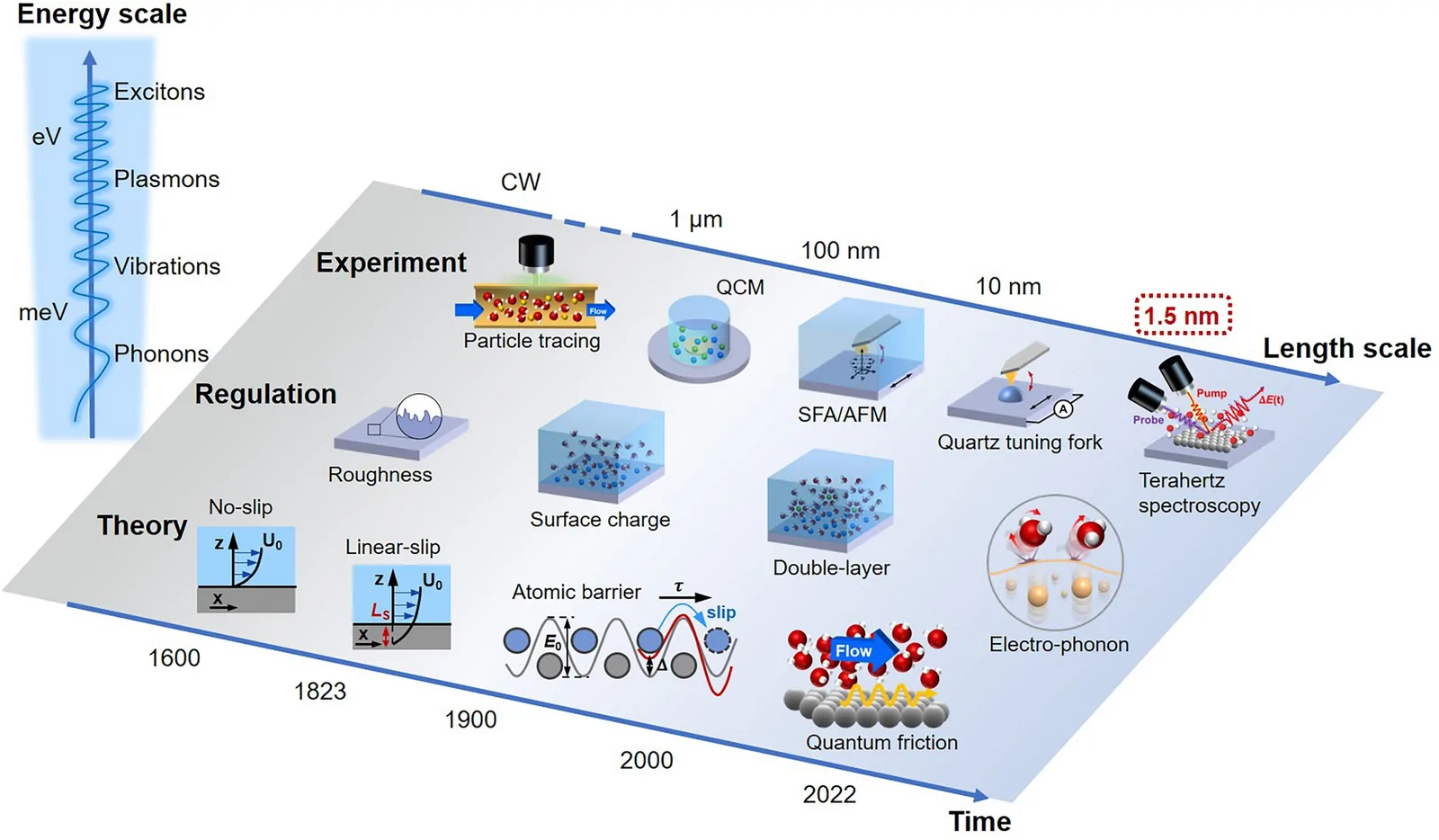
Timeline of the significant advances of solid–liquid friction, covering interfacial theories, regulation, and experimental techniques for probing flow characteristics across length, time, and energy scales. The length scale of 1.5 nm is the critical threshold between continuous media and quantum descriptions. Studies have evolved from the initial no-slip theory to an understanding of linear slip in terms of atomic energy barriers, and further to the discovery of quantum friction mechanisms. The friction modulation has been investigated separately for the surface roughness, surface charge density, electric double layer (EDL), and electro-acoustic coupling. Experimental techniques contain particle tracing, quartz crystal microbalance (QCM), surface force apparatus (SFA), and atomic force microscopy (AFM) developed for spherical models, non-contact tuning-fork AFM, and terahertz spectroscopy

Over the past two centuries, theories of solid–liquid interfacial friction have evolved from macroscopic experiences toward mesoscopic mechanisms. In the classical stage, macroscopic drag-reduction approaches, such as interfacial microstructure design [6, 7] and surface chemical modification [8–10], were developed, with slip length optimized through surface texturing or the introduction of transition interlayers [11–13]. With advances in nanoscale characterization, interfacial wetting behavior [14–16], the electrical double layer (EDL) [17, 18], water molecular ordering [19, 20], and ion adsorption [21] emerged as critical factors influencing solid–liquid friction. Accordingly, scaling-law formulations [22–24], Derjaguin–Landau–Verwey–Overbeek (DLVO) theory [25], and energy barrier models [26–28] were advanced, elucidating quantitative relationships by which surface charge distribution and atomic potential barriers govern friction (Fig. 1) [29–33].

At the macroscopic and microscale, classical continuum theories effectively describe momentum transfer at solid–liquid interfaces under the core assumption that fluids exhibit spatially homogeneous viscosity, density, and a continuously differentiable velocity field. However, non-equilibrium molecular dynamics simulations reveal that when the confinement scale approaches ~ 1.5 nm, the continuum assumption fails [34, 35]. Under these conditions, layered structuring of liquid, steric hindrance, and steep viscosity gradients dominate transport, leading to pronounced divergences in water and ion dynamics [36]. Investigations at this critical threshold further confirm the limitations of classical theories in the sub-nanometer regime [37]. Consequently, elucidating quantum-scale interfacial friction mechanisms becomes essential, as it provides the most fundamental physical description when continuum models fail.

In the early 2000s, the pioneering characterization of carbon nanotubes (CNTs) initiated molecular-scale studies into confined fluid transport [38]. Subsequent experiments revealed that CNTs with diameters of a few ångströms (Å) can efficiently exclude hydrated ions while permitting water molecules to permeate rapidly with ultralow friction [39, 40]. In addition, recent studies have further revealed phenomena beyond classical predictions, including ion selectivity and faster water flow with smaller nanotube diameters (anomalous radius dependence of friction) [41, 42]. These findings highlighted the complexity of solid–liquid interfaces under nanoscale confinement. For example, water molecules have been observed to traverse CNTs (< 100 nm) at extremely high flow rates that exceed classical theory by several orders of magnitude, whereas structurally similar boron nitride nanotubes (BNNTs) did not exhibit comparable behavior [43, 44]. This contrast arises from fundamental differences in electronic structure: Graphitic CNTs contain delocalized electrons that confer electrical conductivity, whereas BNNTs are typical electrical insulators [45, 46]. Consequently, it is increasingly speculated that quantum-scale interactions critically influence nanoscale interfacial friction, highlighting the need to uncover its underlying mechanisms.

Simulation methods are an important tool for studying and understanding quantum-scale friction at solid–liquid interfaces. Molecular dynamics (MD) simulations first demonstrated that the marked difference in the friction coefficient of water on graphene versus boron nitride surfaces is closely related to their electronic structures, advancing the understanding of the critical role of electrons in interfacial friction [47]. In 2022, Bocquet discovered through simulations that Coulomb interactions between flowing water molecules and excited electrons in graphene make a significant contribution to friction, and they defined this electron-dominated phenomenon as “quantum friction” [48, 49]. These theoretical advances challenge the traditional Born–Oppenheimer approximation, in which atomic motion and electronic states are treated independently, and offer a new perspective for explaining the anomalous ultra-low friction of water flow inside CNTs.

In addition, other electron- and phonon-related phenomena at solid–liquid interfaces have been extensively investigated. The widespread phenomenon of contact electrification at solid–liquid friction interfaces was primarily attributed to electron transfer, leading to the widely accepted “electron cloud model” [50, 51]. Further research revealed that energy released at solid–liquid friction interfaces excites and separates a large number of electron–hole pairs, generating measurable triboelectric signals [52]. Lizée et al. reported that the nonmonotonic dependence of slip length on liquid relaxation rate arises from resonant coupling between mica phonons and density fluctuations in the liquid, enabling effective momentum transfer and demonstrating the important contribution of solid phonon excitations to interfacial friction [53]. Collectively, these studies indicate that quantum phenomena such as electron transfer, electron excitation and recombination, and electron–phonon coupling, emerge as key mechanisms governing friction at nanoscale solid–liquid interfaces. Quantum effects may dominate the frictional behavior at solid–liquid interfaces under the following conditions: (a) confinement reaches the nanoscale, enhancing quantum tunneling and interfacial fluctuations; (b) the solid exhibits high electron density or strong phonon excitations, and the liquid is polar or highly dielectric, facilitating quantum-state coupling; and (c) external fields, such as light, electric, or thermal fields, modulate the electronic structure of the solid, making quantum excitations the primary energy dissipation. However, current advancements in this field lack a systematic compilation, which not only impedes deeper theoretical understanding but also limits practical implementation in emerging applications.

**Fig. 2:**
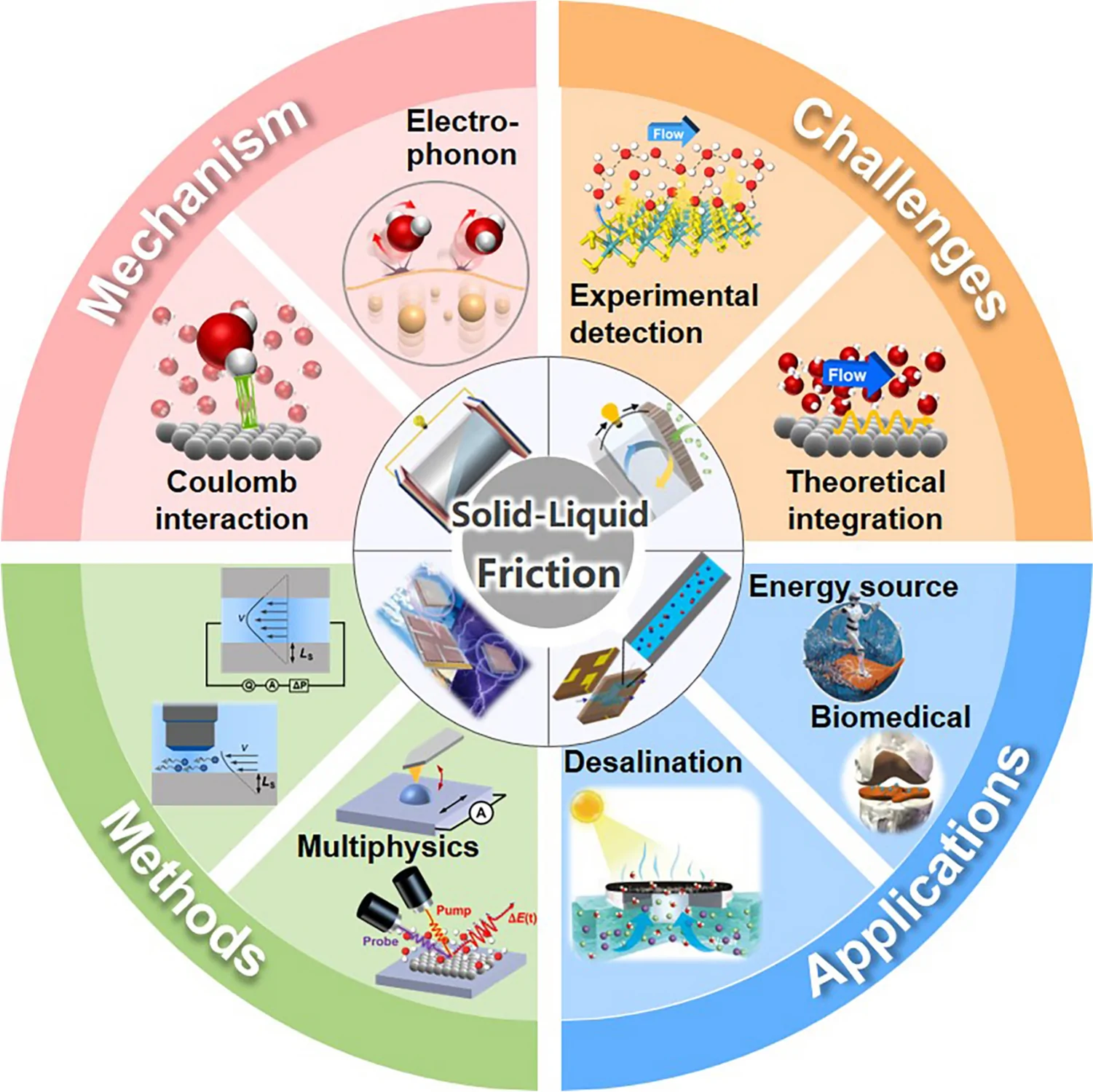
Illustration to the outline of this review, covering methods for quantum-scale solid–liquid friction, core friction mechanisms, emerging applications, and the theoretical and experimental challenges for future studies. Reproduced with permission [54–59]. Copyright © 2022, American Chemical Society. Copyright 2022, Elsevier B.V. Copyright © 2020, American Chemical Society. Copyright © 2019, John Wiley and Sons. Copyright © 2021, John Wiley and Sons. Copyright 2022, Elsevier B.V

To address this, this review provides a comprehensive overview of quantum-scale friction theories at solid–liquid interfaces and their emerging applications. It aims to enhance comprehension of existing theories and technologies, reveal research gaps, and propose future research directions to advance the development of quantum-scale friction. This review first summarizes the core simulation approaches and advanced experimental techniques for probing quantum-scale friction at solid–liquid interfaces, and then systematically discusses the underlying mechanisms revealed by these methods. Emphasis is placed on the behavior of quantum-scale excitations at flowing interfaces and their contributions to friction, and the transformative potential of these insights is assessed for frontier applications such as low-power nanofluidic devices, high-efficiency energy storage systems, biomedical devices, super-lubrication coatings, and next-generation seawater desalination technologies (Fig. 2). Although the theoretical framework has begun to take shape, significant gaps remain in cross-scale validation and sub-nanometer/femtosecond-resolved experimental capabilities. In response, a breakthrough path based on simulation-experiment integration with a focus on application-oriented approaches is preliminarily proposed. Lastly, this review emphasizes the importance of interdisciplinary collaboration across physics, chemistry, and materials science in the study of quantum-scale solid–liquid friction, and discusses the challenges and opportunities associated with translating theoretical findings into engineering applications.

## Theoretical Modeling and Computational Simulations

The limitations exposed by traditional macroscopic and mesoscopic friction theories are the fundamental driver propelling solid–liquid friction research toward the quantum scale. By elucidating coupling mechanisms between electronic excitations and the vibrational modes of fluid molecules, quantum-scale friction theories have provided plausible explanations for the phenomenon of ultra-low-friction transport at nanoscale solid–liquid interfaces and have rapidly become a focal point in the field. On this basis, researchers are systematically advancing the relevant theoretical framework through various simulation methods.

### Molecular Dynamics Simulation Methods

For over a decade, the key role of electronic effects and dynamic processes such as electron transport and charge transfer in solid–liquid friction has been a major research focus, and early conjectures and studies relied primarily on MD simulations. In 2014, Tocci et al. demonstrated by MD that water on graphene and boron nitride surfaces with similar atomic structures exhibits markedly different friction coefficients [47]. These differences were attributed to additional energetic corrugation on BN, arising from specific electron structure effects. This work provided a basis for using MD-based approaches to investigate friction-related electronic effects.

**Fig. 3:**
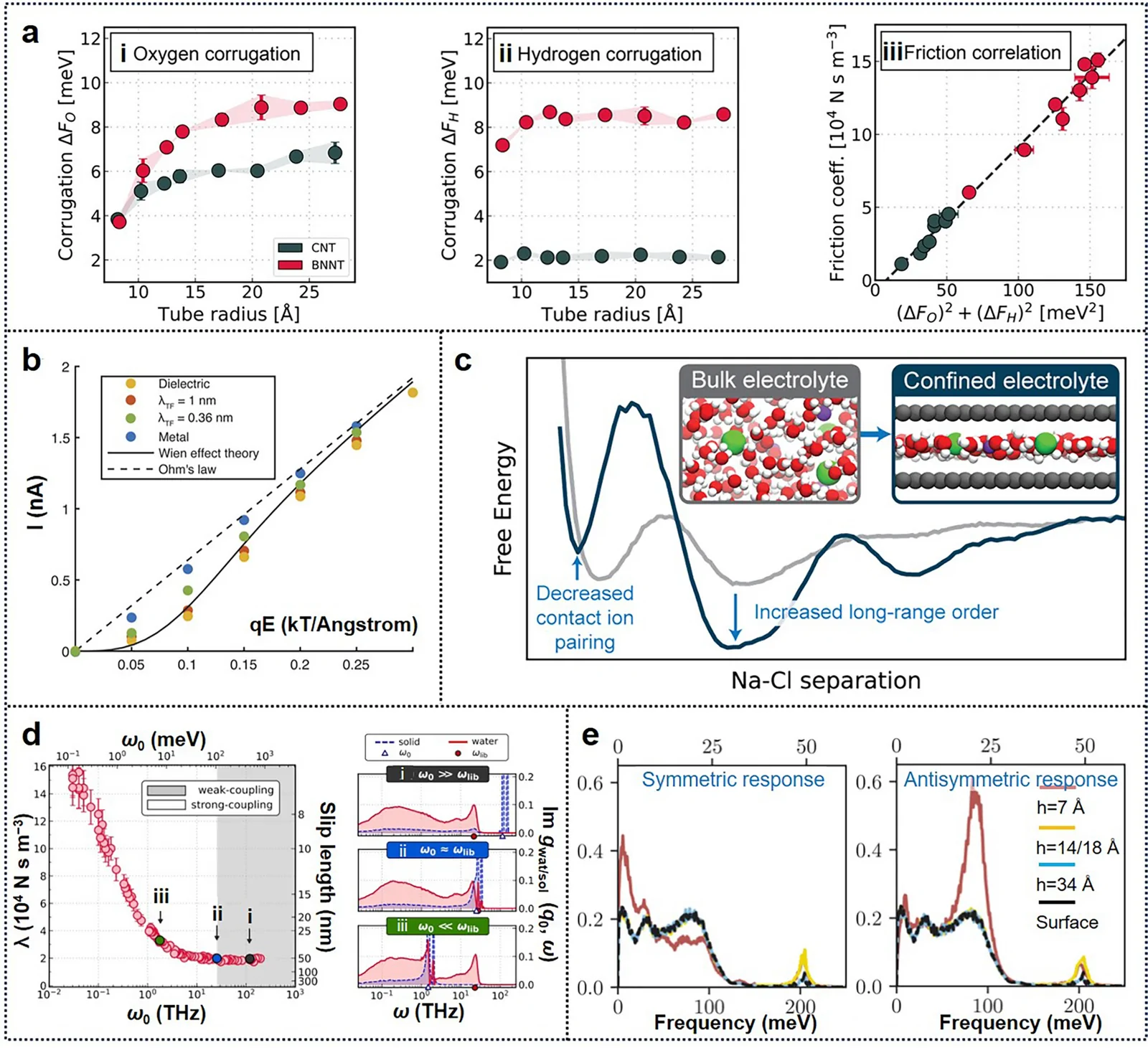
Recent molecular dynamics simulations of solid–liquid interfacial friction. **a** Water friction in CNTs and BNNTs is closely related to the free energy surfaces of oxygen and hydrogen. Reproduced with permission [32]. Copyright © 2022, American Chemical Society. **b** Brownian dynamics simulations of point-like ions constrained to move in two dimensions show Wien effect nonlinearity through effective potential interactions. Reproduced with permission [60]. Copyright © 2022, AIP Publishing. **c** Free energy of ion pairing in highly confined electrolytes deviates substantially from that in bulk solutions, observing decreased contact ion pairing and increased solvent-separated ion pairing. Reproduced with permission [61]. Copyright © 2024, American Chemical Society. **d** When dielectric fluctuations of solid and liquid overlap, the main features of g_wat/sol_(*q*_0_, *ω*) are disturbed, resulting in a significant increase in friction. Reproduced with permission [62]. Copyright © 2023, American Chemical Society.** e** Antisymmetric response functions for different confinements, with extra high response at 7 Å. Reproduced with permission [63]. Copyright © 2024, The Royal Society of Chemistry

Thiemann and colleagues subsequently employed machine-learning-driven MD to reproduce, with first-principles accuracy, the microscopic origins of water transport and frictional differences in CNTs and BNNTs [32]. By analyzing free energy landscapes of water molecules, it was found that the low friction on carbon surfaces originates from a low-energy barrier for oxygen atom migration, which is closely related to the electronic structure of carbon materials. In contrast, the high friction on BN surfaces results from energetic corrugation induced by hydrogen–nitrogen interactions (Fig. 3a). The unique insulating properties of BN materials lead to a different electron excitation pattern, thereby increasing atomic interactions and friction. This study not only provides reliable quantitative values for water transport in defect-free nanotubes but also offers a potential perspective based on intermolecular interaction energies and quantum-scale excitation for quantitatively distinguishing friction differences among low-dimensional materials.

Having explored the microscopic origins of water transport and friction differences in CNTs and BNNTs, recent research has delved into the ionic transport within nanochannels, revealing its close connection with friction. Kavokine developed an MD model of effective Coulomb interactions in nanochannels based on the surface response function [60]. Simulations revealed that when electrolytes in aqueous solution are confined within channels of dimensions comparable to the molecular diameter, the dielectric properties of the channel walls enhance ion–ion Coulomb interactions, a phenomenon referred to as “interaction confinement.” Dynamic simulations further confirmed the feasibility of tuning ion transport through modulation of electronic properties (Fig. 3b). Michaelides extended this approach to highly confined electrolytes and observed pronounced deviations in the free energy of ion pairs relative to bulk solution, closely related to the interplay between the electronic structure of graphene (Fig. 3c) [61]. This phenomenon not only governs the structure of nano-confined electrolytes but also influences friction at the solid–liquid interface, providing indirect evidence for charge transfer mechanisms underlying quantum-scale friction.

Building on this foundation, a classical MD model capable of fine-tuning the dielectric spectrum of solids was employed to confirm the threshold effect of quantum friction [62]. When the solid’s dielectric response overlapped with the molecular degrees of freedom and Debye modes of water, the observed increase in friction closely matched theoretical predictions (Fig. 3d). This finding confirms that molecular frequency-domain resonance matching (spectral matching) at the solid–liquid interface substantially enhances energy transfer, thereby increasing friction. Kavokine further extended the conventional surface response function to two-dimensionally confined systems by introducing confined response functions to describe Coulomb interactions between solid walls and liquids (Fig. 3e) [63]. The MD model revealed that traditional continuum theories underestimate the enhancement of electronic polarization at ion–wall interface arising from confinement effects. This highlights the necessity of additional physical corrections at the nanoscale to account for quantum-scale interactions, such as electron behavior, and provides critical dielectric constraints for refining the boundary conditions of friction.

These studies have surpassed the limitations of traditional continuum models by extending the research scale to the level of electron–molecule coupling, revealing that interfacial behavior is essentially a cooperative manifestation of quantum effects and classical statistics at the mesoscopic scale. However, MD simulations, which rely on classical force fields to describe electron–molecule interactions, often oversimplify processes such as dynamic electron cloud polarization and transient charge transfer, potentially leading to misinterpretation of interfacial friction.

### Quantum Mechanical Computational Methods

**Fig. 4:**
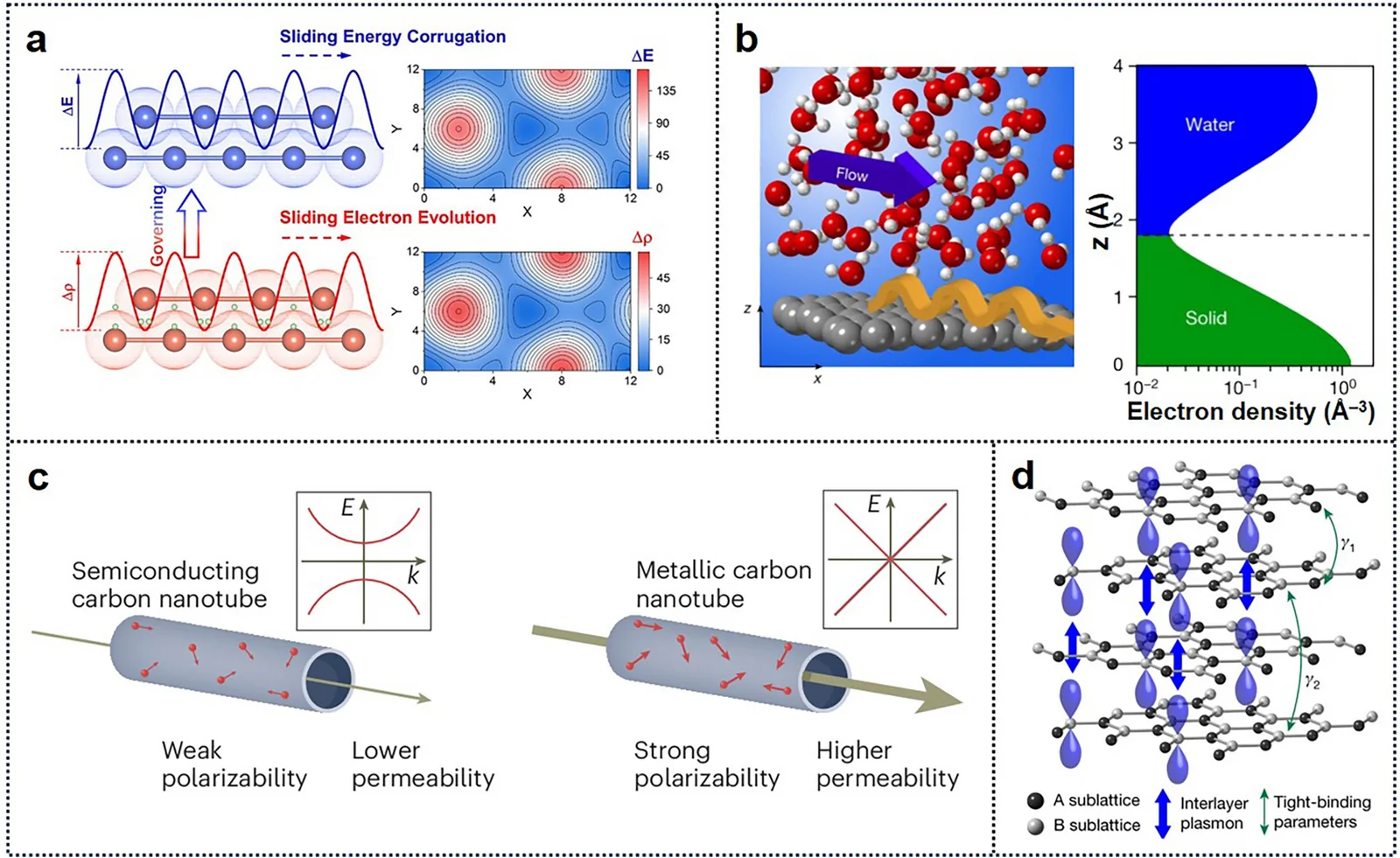
Other advances in simulations of solid–liquid interfacial friction.** a** Sliding energy landscape Δ*E* and corresponding charge density evolution Δ*ρ* in slip. Reproduced with permission [64]. Copyright © 2023, American Chemical Society.** b** Average electronic density at the water–graphene interface obtained from density functional calculations. Reproduced with permission [49]. Copyright © 2022, Springer Nature. **c** Weakly polarizable semiconducting CNTPs exhibit lower water permeability than the strongly polarizable metallic CNTPs. Reproduced with permission [70]. Copyright © 2024, Springer Nature. **d** Plasmonic mode exhibiting the strongest coupling between graphite and charge fluctuations in water, arising from electron jumping between graphene layers. Reproduced with permission [49]. Copyright © 2022, Springer Nature

Compared with classical molecular dynamics, first-principles calculations are uniquely suited to resolving electronic states and are indispensable tools for revealing electronic effects in interfacial friction. Sun employed density functional theory (DFT) to systematically reveal universal trends across van der Waals, metallic, ionic, and covalent frictional interfaces: the evolution of electronic density along a sliding path is synchronized with the energy landscape, establishing a clear linear dependence of frictional dissipation on the electronic response (Fig. 4a) [64]. Combining DFT simulations with experiments, it has been further demonstrated that defects in van der Waals heterostructures modulate charge density evolution and thereby increase frictional energy barriers [65]. These studies not only confirm the generality of electronic degrees of freedom in friction regulation but also emphasize the irreplaceable role of DFT in resolving interface electronic structure, particularly for capturing correlations between ground-state electron properties and atomic configurations.

Building on these strengths, DFT-based approaches have also achieved substantive progress in investigating the contribution of electrons to friction in solid–liquid systems. Kim used density functional theory in classical explicit solvents (DFT-CES) to model interfacial interactions that include both van der Waals and electrostatic contributions, highlighting the key role of the solid surface electronic structure in solid–liquid friction [66]. Subsequently, DFT-driven ab initio molecular dynamics (AIMD) was applied to accurately describe weak interactions between water and two-dimensional (2D) materials such as graphene, boron nitride, and molybdenum disulfide (MoS_2_) [33]. It was proposed that interfacial friction is determined primarily by the first-atomic layer. The underlying mechanism is that the electron clouds of the first layer atoms modulate the long-range electrostatic and van der Waals forces between the solid surface and water molecules, thereby effectively regulating the interfacial friction.

As the low-friction property of the water–graphene interface has been widely recognized in research, DFT was employed to compute the average electronic density at the water–graphene interface, initially revealing that dynamic polarization of interfacial electronic states contributes critically to solid–liquid friction (Fig. 4b) [49]. Bocquet and co-workers utilized DFT to analyze the charge-response functions of confined water and to quantify the dynamic response of Coulomb potentials between solid walls and confined liquids [63]. The pronounced confinement effects in the charge fluctuation modes of water in nanochannels were found, especially when the channel width was reduced to ~ 7 Å. These effects modify Debye and hydrogen-bond stretching modes, and directly affect quantum friction contributions and energy transfer, thereby confirming the important role of electronic density distributions in frictional behavior.

However, existing DFT models are constrained by the adiabatic (Born–Oppenheimer) approximation, principally describing static electronic polarization and failing to capture femtosecond-scale transient charge transfer processes. Moreover, DFT treatments of large systems (> 1000 atoms) or long-range van der Waals interactions still rely on approximations that can compromise the accuracy of predictions for water–interface friction. Consequently, developing a unified description that integrates electronic quantum dynamics (e.g., tunneling) with classical statistical behavior of fluids (e.g., molecular diffusion) remains challenging. There is an urgent need to develop multi-method linkage strategies: for example, determining whether real-time or time-dependent DFT should be incorporated to capture femtosecond-scale electronic rearrangements, and identifying how cross-scale parameter coupling schemes can be established to bridge quantum fluctuations and friction properties.

### Multi-Scale Simulation Methods

The reliability of solid–liquid friction theory depends on cross-validation among multiple methods, as single-scale simulations cannot fully capture interfacial dynamics. Consequently, coupling tools across scales has become essential for resolving quantum behaviors at friction interfaces.

Under extreme confinement below 1 nm, molecular transport exhibits single-molecule occupancy characteristics, and bulk properties effectively disappear, rendering interfacial effects predominant. Due to the limited number of molecules available for statistics, conventional ensemble-averaged parameters, such as viscosity, velocity profiles, and residence times, cannot be reliably extracted. Recently, Kim et al. proposed an ergodic-sampling framework that compensates for spatial averages through temporal averaging, enabling the reconstruction of macroscopic transport parameters from sparse single-molecule trajectories [67, 68]. This approach not only demonstrates high stability in CNTs and sub-nanometer channels, but also provides a verifiable numerical pathway to investigate quantum-scale friction mechanisms under extreme confinement.

A representative work by Bocquet combined molecular dynamics capturing the thermal motion and density fluctuations of water with quantum mechanical treatments of graphitic electronic states and charge fluctuations to investigate the origin of friction at water–carbon interfaces (Fig. 4c) [49, 69, 70]. This approach directly captured the coupling between charge density fluctuations in graphitic materials and the Debye modes of water, revealing correlations that single-scale simulations fail to access.

Franzese further emphasized the necessity of treating water as a many-body system in such multiscale schemes—that is, focusing on collective behavior rather than a simple sum of single-molecule properties [71–73]. The characteristic frequencies of both graphene plasmons with free electrons as the carriers and aqueous Debye modes can lie in the terahertz range, enabling momentum transfer under resonance (Fig. 4d). In this regime, the Born–Oppenheimer approximation is broken down, and strong coupling can induce electron hopping within graphitic layers, thus increasing friction. This underscores the indispensable role of multiscale simulations in elucidating the quantum-scale mechanism of solid–liquid friction.

Classical MD captures molecular motion but cannot describe electronic behavior governed by wave–particle duality and quantum tunneling. Quantum mechanical simulations excel at resolving electronic states, yet typically neglect non-equilibrium excitation processes are computationally expensive, posing challenges for large, long-time solid–liquid systems. Multiscale simulation approaches offer unique advantages in investigating interfacial electron–water coupling. By integrating quantum–mechanical calculations with classical MD simulations, both the behavior of electrons and the long-range dynamics of water molecules can be captured simultaneously, thereby achieving accurate coupling between quantum effects and molecular motion. For example, at graphene–water interfaces, multiscale simulations have revealed resonant coupling between excited electrons and water molecules, providing theoretical support for quantum-scale friction.

Nevertheless, several challenges remain. First, most existing models rely on simplified assumptions such as ideal 2D materials, whereas real surfaces exhibit defects, oxidation, and charge fluctuations that can significantly affect the universality of outcomes [31, 74]. Second, the direct correlation between physical quantities across scales, such as electron transfer and water molecule diffusion, has yet to be fully elucidated. In addition, femtosecond electronic processes are difficult to validate experimentally, hindering model validation and improvement. To bridge the gap between theory and experiment, stochastic modeling and experimental-feedback schemes could be employed. For instance, Monte Carlo sampling and stochastic dynamics enable quantitative characterization of random perturbations. Meanwhile, by integrating quantum–mechanical parameters from time-dependent density functional theory (TDDFT), mesoscopic transport parameters from non-equilibrium Green’s function (NEGF), and macroscopic rheological parameters, a closed-loop system of “stochastic process modeling-computational prediction-model iteration” can be established. This approach can reduce the interference of random variables, thereby enabling in-depth analysis of the multi-physical coupling phenomena at interfaces.

## Experimental Techniques and Advancements

Although a theoretical framework for quantum-scale friction has been established, its experimental validation remains challenging. One reason is that quantum signals at solid–liquid interfaces can easily be drowned out by classical interfacial noise. Another is the requirement for simultaneous femtosecond temporal resolution and nanometer-scale spatial precision. Recent interdisciplinary innovations in ultrafast spectroscopy, nanoprobing, and nanofluidics have propelled research from indirect observation toward direct dynamic investigation.

### Energy Transfer Measurements Based on Terahertz Spectroscopy

Terahertz (THz) spectroscopy, which spans from femtoseconds to nanoseconds, has become a sensitive tool for probing fluctuations in the structure and dynamics of solid–liquid interfaces. Its applications range from studies of excitons and Cooper pairs in solids to investigations of biomolecular hydration dynamics and quantum friction at solid–liquid interfaces [75]. For instance, THz spectroscopy has been used to reveal that monovalent cations (Li⁺, Na⁺, etc.) alter the dynamics of surrounding water molecules, with a fast relaxation process in water [76]. This study provided frequency-domain evidence for the collective motion of water molecules—a prerequisite for quantum friction. In another example, THz–IR absorption combined with sum-frequency generation (SFG) spectroscopy was used to characterize the 2D hydrogen-bond network at the interface between water and a weakly interacting material [77]. The competition between water–surface and water–water interactions was quantified. It was demonstrated that a characteristic peak in the THz range (around 160 cm⁻^1^) can serve as a direct spectral marker of the 2D hydrogen-bond network, providing a water-molecular perspective of solid–liquid interactions in friction.

**Fig. 5:**
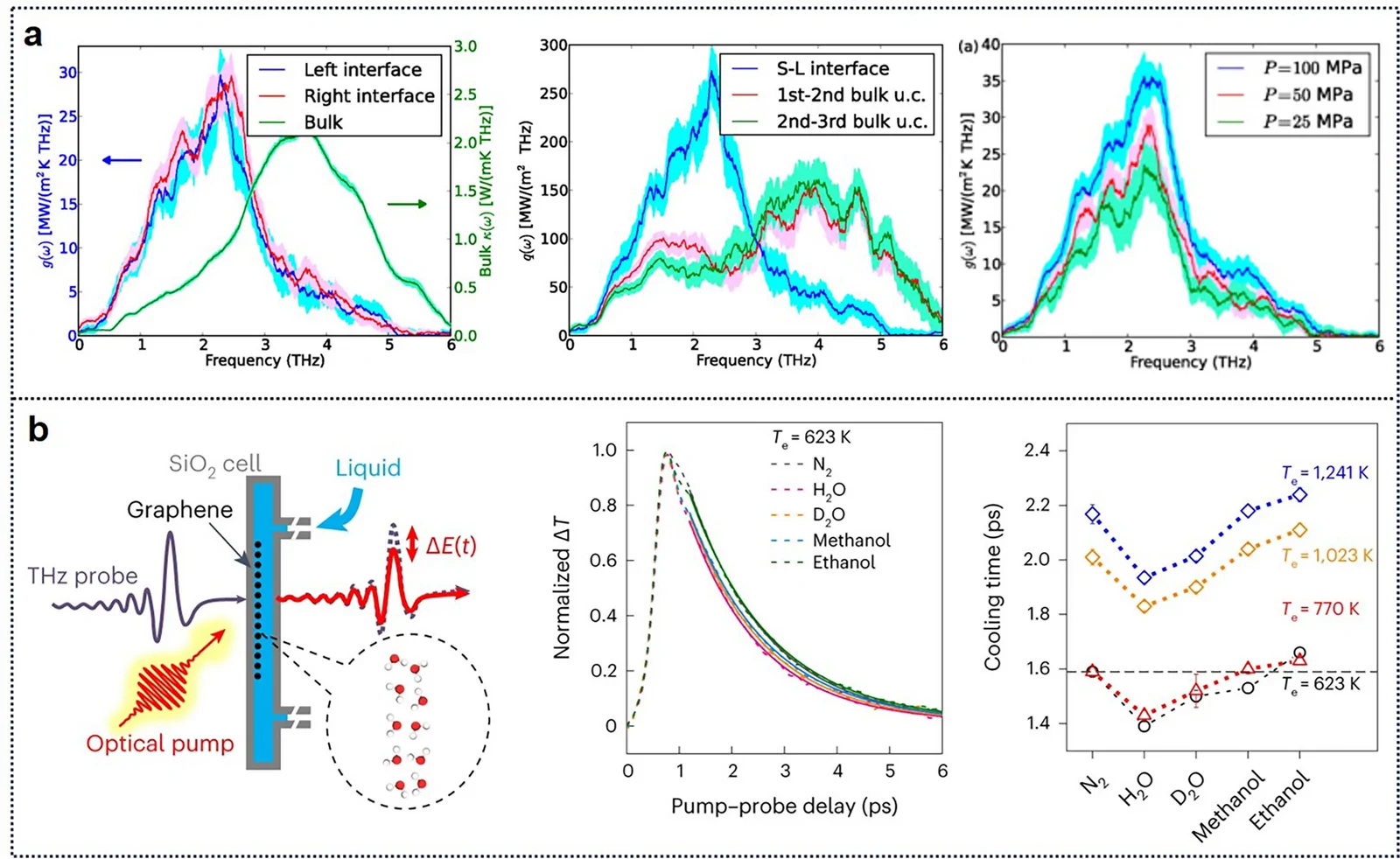
Recent advances in experiments of quantum friction at solid–liquid interfaces.** a** Spectral conductance versus frequency at the liquid–solid interface. Reproduced with permission [78]. Copyright © 2016, American Physical Society. **b** Measurement of picosecond hot electron relaxation in graphene. Normalized electron temperature as a function of time suggests faster electron cooling at the water interface. Reproduced with permission [79]. Copyright © 2023, Springer Nature

Moreover, THz techniques have also been increasingly applied to the analysis of solid–liquid energy transfer processes. Sääskilahti performed a spectrally decomposed heat-flux analysis and confirmed that surface modes at the boundary of the Brillouin zone dominate energy transfer and dissipation at solid–liquid interfaces (Fig. 5a) [78]. This finding laid the foundation for subsequent precise characterization of electron, phonon, and associated friction mechanisms using THz spectroscopy. Furthermore, energy transfer related to quantum friction was first quantified using THz time-domain spectroscopy (THz-TDS) [79]. By constructing the theoretical model, it was predicted that in-plane plasmon polariton in graphene can mediate electron–water coupling, with a strength weaker than that in graphite. The results indicated that, compared to methanol or ethanol, the water medium accelerates the thermal relaxation of the graphene electron cloud—a specific energy transfer channel associated with quantum friction (Fig. 5b). This difference is attributed to the synergistic interaction between hydrogen-bond network of water and electron–phonon coupling, providing frequency domain evidence for the collective polarization behavior of water molecules in quantum friction.

While Terahertz spectroscopy successfully captures the energy transfer signature of quantum friction, it cannot distinguish the contributions of quantum friction and classical heat conduction to the electron cooling rate. Therefore, more refined control experiments are needed. For example, isotopically substituted water such as D_2_O could be used to modulate the vibrational frequency of hydrogen bonds, thereby highlighting the influence of quantum excitations. Additionally, the quantum coherence of interfacial energy transfer could be tracked in real-time by combining surface plasmon resonance probes.

### Electron–Phonon Measurements with Non-Contact Atomic Force Microscopy

It is widely accepted that electrons and phonons play crucial roles in solid–liquid interface friction at the microscopic scale. Non-contact atomic force microscopy (NC-AFM) with sub-nanometer spatial resolution has emerged as a core tool for resolving electron–phonon coupling and energy transfer at dynamic solid–liquid interfaces.

**Fig. 6:**
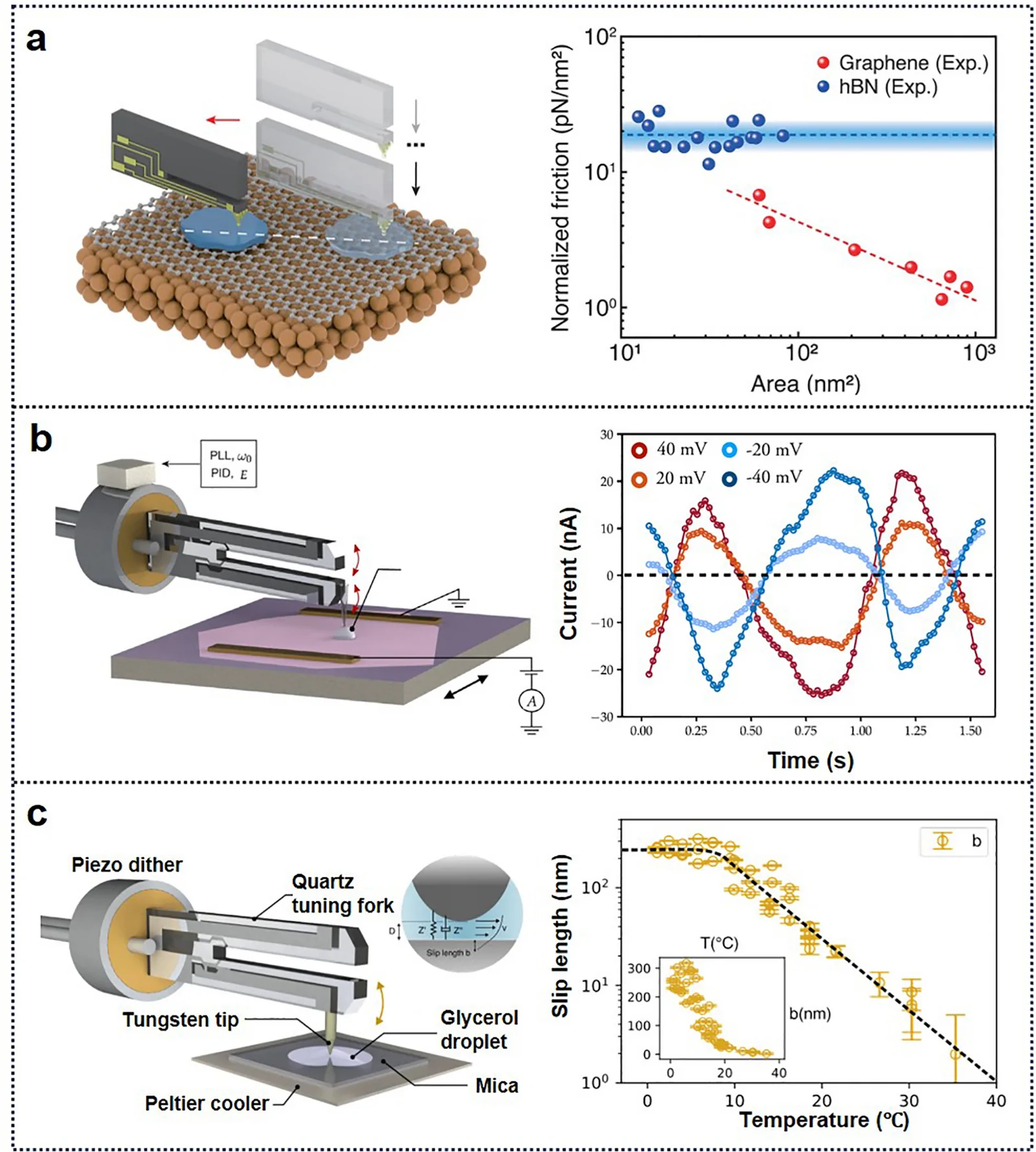
Non-contact AFM measurements. **a** Significant differences in the normalized maximum static friction of water sliding on graphene or hBN substrates. Reproduced with permission [82]. Copyright © 2024, American Association for the Advancement of Science. **b** Periodic variation with time of the current produced by droplet oscillations at different bias pressures in a quartz tuning-fork test. Reproduced with permission [85]. Copyright © 2023, American Physical Society. **c** Tuning-fork AFM system for measuring slip length of water on mica as a function of temperature. Reproduced with permission [53]. Copyright © 2024, Springer Nature

Jiang et al. introduced the qPlus sensor into scanning probe microscopy, markedly improving the spatial resolution for observing interfacial water molecules [80, 81]. Comparison of 2D water films on graphene and BN revealed that the pronounced difference in friction could be attributed to commensurability between water islands and the graphene lattice, as well as to differences in surface electrostatics (Fig. 6a) [82]. Using a similar qPlus-type NC-AFM, Wang et al. observed a large energy dissipation peak within ~ 2 nm of the graphite surface that was inconsistent with phonon dissipation, indicating a transition in the dissipation mechanism to hysteretic processes involving probe vibration and lattice deformation [83]. Recently, the team reported a strong linear relationship between friction and the dissipation rate of interlayer charge transfer in WS_2_/graphene heterostructures, providing direct evidence of friction-induced electron transfer [84].

Building on NC-AFM techniques, Bocquet and Siria developed a tuning-fork AFM that enabled the first experimental measurement of momentum transfer of quantum friction at solid–liquid interfaces (Fig. 6b) [85]. Experiments detected electronic currents of ~ 10 nA induced by micrometer-scale droplet displacements on graphene, several orders of magnitude larger than previously reported frictional currents at carbon–water interfaces [86]. The current magnitude was found to correlate with topological defects associated with surface corrugation. Since current intensity indirectly reflects the friction and defects induced by surface wrinkles directly regulate the phonon excitation efficiency, indicating that quantum friction is closely related to phonon excitation. At the glycerol–mica interface, related experiments using similar instrumentation revealed anomalous dependencies of surface friction on relaxation rates in viscous fluids (Fig. 6c) [53]. Upon cooling, the slip length of glycerol on mica increased by two orders of magnitude, whereas at elevated temperatures the solid–liquid friction exhibited a nonmonotonic dependence on molecular relaxation rate. Such behavior further supports the effective contribution of resonant coupling between solid phonons and liquid density fluctuations to friction and implies that phonons effects should not be neglected.

Although these NC-AFM-based techniques have enabled in situ measurements of signatures from quantum friction, their spatial resolution (micrometer-scale) remains insufficient to resolve single-molecule quantum-tunneling events, and concurrent electron and phonon dissipation channels cannot be disentangled. Recent STM-coupled spectroscopic studies have demonstrated the capability to simultaneously resolve local electronic structure and phonon excitation modes, enabling the identification of energy transfer pathways across friction interfaces at the quantum scale [87, 88]. This provides a foundation for probing coupling mechanisms, such as electron–phonon and electron–liquid interactions, using the STM technique.

### Electrical Property Measurements Based on Nanofluidic Devices

While THz spectroscopy and non-contact atomic force microscopy have enabled microscopic characterization of quantum friction at solid–liquid interfaces, macroscopic transport measurements remain essential for establishing cross-scale correlations between quantum-scale excitations and solid–liquid friction [89]. This behavior arises from the significant influence of quantum effects on solid–liquid friction under nanoscale constraints, causing interfacial dynamics to frequently deviate from classical theory. Transport measurements provide critical insights into the origins of such deviations.

**Fig. 7:**
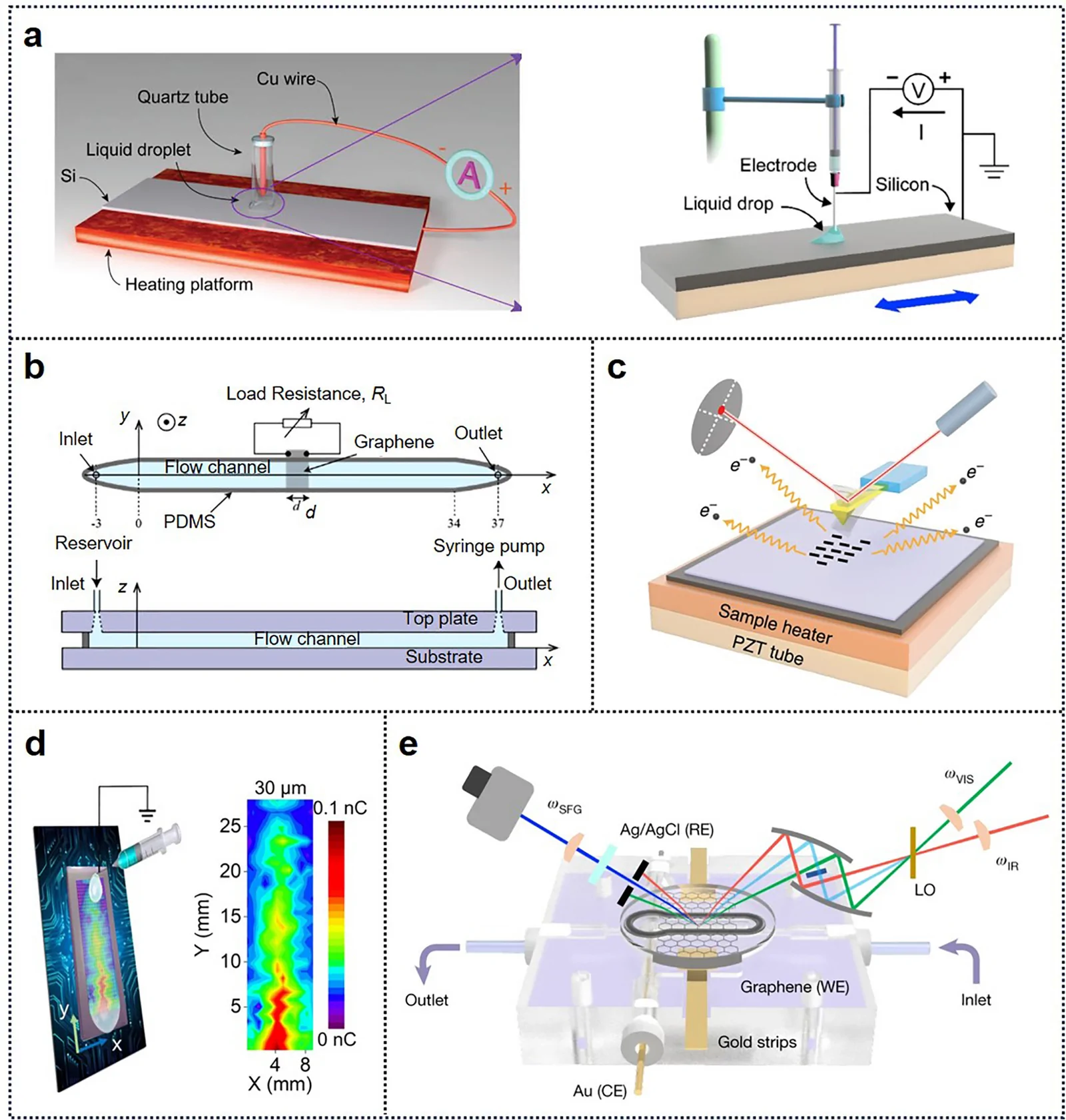
Measurements based on nanofluidic devices and dynamic charge imaging. **a** Experimental setup of tribo-voltaic effect at the water–silicon interface. Reproduced with permission [52, 90]. Copyright © 2021, John Wiley and Sons. Copyright 2020, Elsevier B.V.** b** Microfluidic chip based on graphene–water interface, showing a correlation between the flow conditions of the liquid medium and graphene conductivity. Reproduced with permission [96]. Copyright © 2024, AIP Publishing. **c** SPM platform for the electron transfer measurement. Reproduced with permission [98]. Copyright © 2020, Springer Nature.** d** Sliding water droplet induces charge transfer between the solid and liquid, with the electrode array measuring the induced charge at each point. Reproduced with permission [100]. Copyright © 2023, American Chemical Society. **e** In situ electrochemical SFG device for precise probing of molecular orientations at flowing graphene–water interfaces. Reproduced with permission [103]. Copyright © 2024, Springer Nature

With rapid advances in nanofluidic device fabrication and measurement techniques, fluid transport experiments have become a key tool for revealing microscopic friction mechanisms. Wang et al. developed a fluid transport measurement system combining a quartz tube–copper electrode sliding apparatus with a voltmeter, and demonstrated that temperature-induced changes in contact angle (from 86.72° to 60.36°) at the friction interface were accompanied by enhanced electrical output. These findings provided experimental evidence for a friction mechanism, whereby the formation and dissociation of interfacial chemical bonds releases energy to excite electron–hole pairs [52]. This quantum-scale excitation process is fundamentally coupled with macroscopic friction at the solid–liquid interface. In a related study, a silicon-based droplet sliding apparatus controlled by electrode probes (Fig. 7a) was constructed to reveal the regulatory effects of sliding velocity, contact area, and salt concentration on triboelectric signals, offering macroscopic transport evidence for charge transfer mechanisms underlying quantum-scale friction [90]. Specifically, as a macroscopic manifestation of interfacial charge separation and transfer, the measured triboelectric signal is closely correlated with increased friction, indirectly suggesting that quantum-scale excitations may constitute the fundamental physical origin of friction dissipation.

Similar transport experiments are often employed in nanofluidics to explore friction-related interfacial phenomena. For instance, the conductivity of single-walled carbon nanotubes (SWCNTs), as well as the quantitative relationships between ionic current, water permeability, and solid–liquid friction, have been measured and optimized [91, 92]. Xie et al. reported that, in single graphene nanochannels, the water slip length varied from 0 to 200 nm and was independent of channel height, attributing to surface charge and substrate effects [93]. Giem et al. fabricated 2D slits with heights of only a few ångströms and observed ion-specific friction: water flow resistance dropped sharply, Na⁺ and Cl⁻ permeation became undetectable, and only H⁺ could diffuse through a monolayer of water [94]. Under pressure and an external electric field, a bias of only a few tenths of a volt was shown to enhance pressure-driven ion transport by up to 20-fold [95]. Tanemura et al. correlated graphene conductivity with water flow conditions in nanochannels, revealing that the spatial transition from irregular to laminar flow critically affects conductivity [96] (Fig. 7b). This phenomenon stems from flow-induced quantum excitations at the graphene–water interface and serves as a core carrier of solid–liquid friction energy dissipation.

These findings deviate from predictions of the classical Poiseuille flow model. By precisely controlling confinement dimensions and interfacial properties, the modulation of solid–liquid friction can be traced back to the electron and molecule level, thereby bridging classical tribology and quantum friction theory.

### Macroscopic Dynamic Charge Imaging Techniques at Solid–Liquid Interfaces

Conventional characterization of solid–liquid interfaces has largely focused on static electrostatic potential measurements at the microscale. However, during dynamic processes such as droplet sliding, charge imaging at the macroscale is crucial for elucidating charge transfer mechanisms and the origins of friction, and is increasingly recognized as a key approach for investigating friction at solid–liquid interfaces.

Scanning probe microscopy (SPM) has emerged for probing charge transfer and interfacial potential barriers. By detecting variations in tip–interface interaction or electric potential, SPM enables the visualization of electric double-layer structures and the formation, migration, and evolution of charge adsorption sites [97]. As a specialized form of SPM, Kelvin probe force microscopy (KPFM) enables the measurement of solid–liquid electron transfer based on contact potential difference (Fig. 7c) [98]. Recent studies have combined KPFM with solar illumination during droplet sliding to reveal intrinsic correlations among charge accumulation, dissipation, and fluid adhesion at dynamic interfaces, across scales from millimeters to centimeters [99]. In addition, Zhang et al. recently employed a pixelated electrode array to achieve millisecond-level 2D imaging of transferred charges during liquid sliding (Fig. 7d) [100]. This advancement effectively compensates for the temporal resolution limitations of conventional techniques, providing experimental support for charge generation, migration at solid–liquid friction interfaces. More recently, fluorescence probes combined with confocal microscopy and charge measurements have enabled high-spatiotemporal-resolution imaging of sliding-induced charge redistribution on hydrophobic surfaces [101, 102]. This technology provides an intuitive and quantitative macroscopic approach for studying charge transfer at solid–liquid interfaces, thereby offering critical support for understanding friction phenomena at the quantum scale.

In summary, among the current techniques employed to probe solid–liquid friction at the quantum scale, THz spectroscopy opens a new window onto interfacial dynamics, while NC-AFM offers high-resolution imaging and force measurement capabilities. By linking microscopic friction phenomena with macroscopic fluid behavior, fluid transport measurements further complement the experimental framework. The resolution, features, and applicability of these approaches to interfacial phenomena are systematically summarized in Table 1.

**Table 1:** Comparison of experiment technique for solid–liquid friction

Technique	Basis and resolution	Measured value	Accessible properties and effects	Technical characteristics	Samples
THz Spectroscopy [79]	0.01 THz	Electron cooling rate; electro–phonon interaction	Response of quantum friction to low-frequency vibrations	Detectable low frequency vibration; Low-T to RT; UHV; Atmosphere control	2D materials
Non-contact atomic force microscope [53]	AFM pN-nN	Complex mechanical impedance; friction coefficient	Slip behavior of fluids on solid surfaces	High quality factor; Low-T to RT; Vacuum (1 mbar); Atmosphere control	Soft Matter; biological samples; quartz; mica, etc
Transport Measurement [44, 104, 105]	Flow rate accuracy nL/min; Pressure drop accuracy 0.01 Pa; Slip length accuracy 1 nm;	Fluid pressure drop; transmittance; volume flow rate; ionic current	Low-friction interfacial transport properties; nanolimited domain flow behavior; slip length; surface charge properties	RT-100 °C	Nanotube; low-dimensional materials; hydrophilic/hydrophobic surfaces

Low-T: low temperature; RT: room temperature; UHV: ultra-high vacuum

However, all experimental techniques currently employed in solid–liquid quantum friction research face inherent core challenges:

1.**Nanomaterial fabrication constraints**. Producing nanotubes or other nanomaterials with precise structures (e.g., single-walled CNTs with diameters < 10 nm or chirality-pure structures) remains technically demanding, limiting the construction of standardized experimental platforms and systematic studies [106, 107].

2.**Spatiotemporal resolution limits**. Electron dynamics and friction dissipation under water flow occur at sub-nanometer spatial scales and on picosecond–femtosecond time scales, requiring extremely high-resolution measurements. Techniques such as the THz spectroscopy and ultrafast time-resolved spectroscopy have shown promise for detecting interfacial energy transfer and electronic dissipation [108–110]. However, achieving simultaneous acquisition of multidimensional data, including electron dynamics, fluid flow, and friction, under precisely controlled temperature, pressure, and chemical environments remains highly challenging.

3.**Mismatch between theory and experimen**t. Uncertainties in theoretical models of quantum-scale friction directly constrain experimental interpretation and the design of targeted measurements.

Addressing these challenges will require the development of in situ, multiparametric, synchronous detection techniques, such as integrating X-ray absorption spectroscopy to monitor the evolution of interfacial electronic state or combining neutron scattering to probe dynamic reconstruction of the hydrogen-bond network under shear flow. Such approaches would enable simultaneous tracking of molecular arrangement, electron transport, and phonon coupling. New opportunities may also emerge from ultrafast interfacial imaging techniques, such as pump–probe spectroscopy [111–113], and nonlinear optical approaches such as sum-frequency spectroscopy (Fig. 7e), which have been extensively applied to water interface studies [103, 114, 115].

## Quantum-Scale Mechanisms for Solid–Liquid Friction

By synthesizing relevant simulation methods and experimental techniques, the available tools for investigating quantum-scale friction at solid–liquid interfaces have been clarified. These methods not only reveal the energy dissipation and dynamic behavior at solid–liquid interfaces, but also provide critical evidence supporting the theory of quantum-scale friction.

Classical friction theories, such as the Amontons–Coulomb law and Bowden–Tabor adhesion theory, primarily attribute friction to surface roughness, fluid viscosity, and mechanical adhesion, and have successfully explained macroscopic sliding and hydrodynamic lubrication. However, when confinement approaches the critical molecular scale (~ 1.5 nm), these assumptions break down fundamentally [116, 117]. Continuum-based Sampson formulations and slip models require atomic-scale modifications to account for the coupling between water molecular structural fluctuations and interfacial dissipation [36, 118]. Under such conditions, interfacial friction becomes strongly influenced by electron and phonon excitations in the solid, as well as dipolar vibrations in the liquid, marking a transition from continuum to quantum-scale friction mechanisms. For instance, recent studies have demonstrated that confined water can substantially broaden the vibrational spectrum of graphene, induce charge polarization and redistribution, and consequently alter the friction coefficient by up to 1–3 times [119–121]. These observations highlight a theoretical shift from geometry-dominated classical theories to quantum-scale frameworks, enabling ultrafine control over interfacial friction. In this section, the fundamental mechanisms of quantum-scale friction were systematically discussed, focusing on electron transfer, electronic excitation and recombination, and electron–phonon coupling.

### Electron Transfer and Redistribution

#### Charge Transfer Mechanisms at Solid–Liquid Interfaces

Charge transfer at solid–liquid interfaces is a non-classical phenomenon widely observed in frictional processes, involving the coupling of multiple physicochemical pathways. It is primarily governed by three mechanisms: electron transfer, ion transfer, and the two-step model of EDL formation. Early investigations predominantly attributed charge transfer at solid–liquid interfaces to ion transfer. For example, when water flows across graphene, dynamic variations between the two interfacial equivalent capacitive induce directional ion transport, generating a voltage known as the streaming potential [86]. Traditional electrokinetic models consider this streaming potential as the primary source of charge transfer at solid–liquid interfaces.

**Fig. 8:**
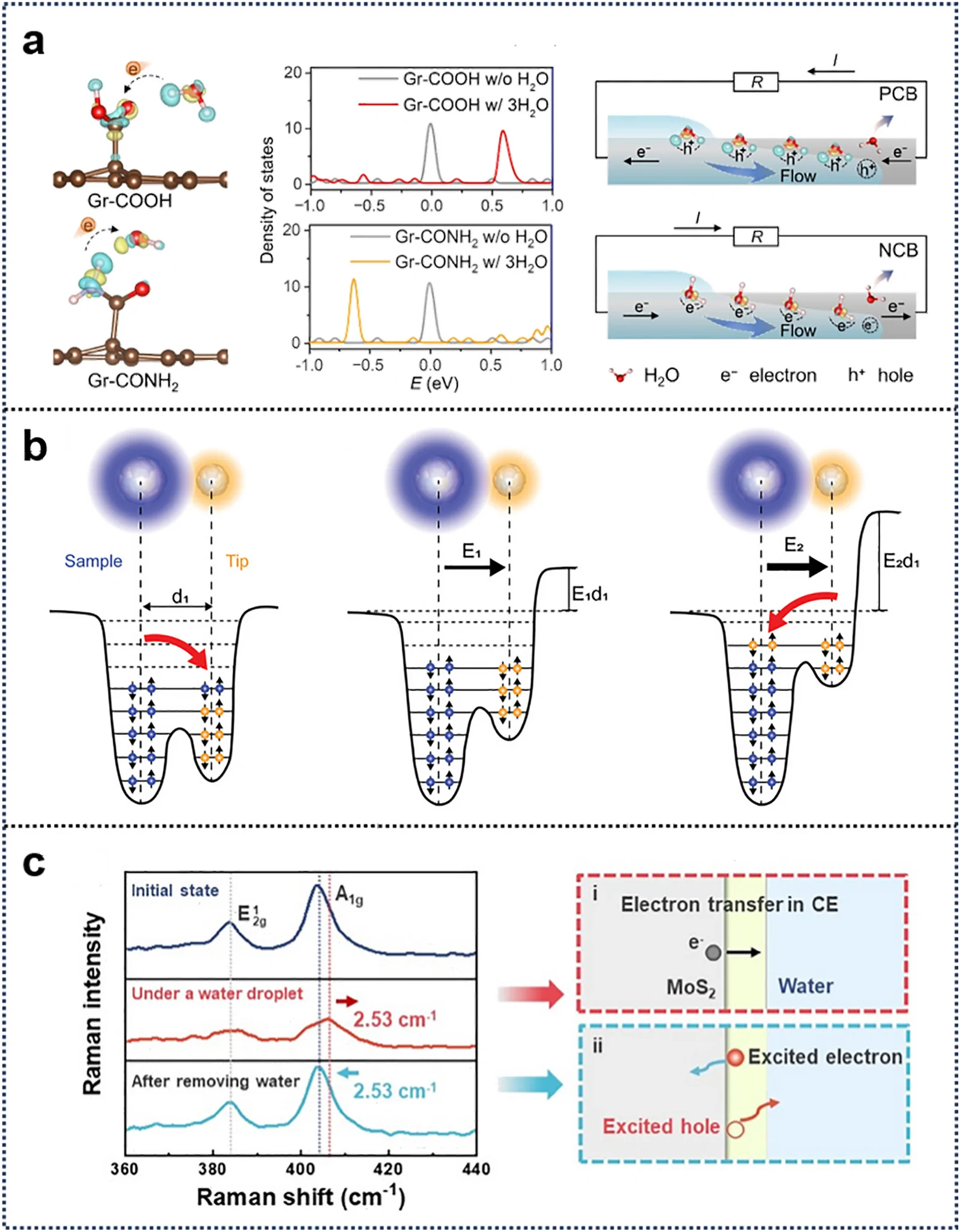
Mechanism of electron transfer at solid–liquid friction interface. **a** Evaporation-driven charge transport model [123]. Copyright © 2024, Tsinghua University Press. **b** Solid–liquid contact electron cloud overlap modeling. Reproduced with permission [130]. Copyright © 2020, John Wiley and Sons. **c** Evidence of double electron transfer under water droplets by Raman spectroscopy of monolayer MoS_2_. Reproduced with permission [134]. Copyright © 2025, John Wiley and Sons

However, studies reveal that at interfaces formed with deionized water, electron transfer and redistribution serve as the primary mechanisms governing interfacial charge and frictional behavior [98, 122]. Recent experiments further show that in partially wetted carbon-black films, the streaming potential accounts for only about half of the measured voltage, indicating the presence of additional charge transfer pathways [123]. Accordingly, an evaporation-induced charge transfer mechanism has been proposed (Fig. 8a). In the wetted region, interactions between water molecules and the substrate induce charge doping and a carrier-concentration gradient. Capillary flow generated by water evaporation drives holes toward the interface, while electrons migrate through the external circuit and eventually recombine with evaporated water molecules, resulting in a sustained electrical output.

In addition, electron transfer between surface functional groups and water molecules has been identified as another major contributor to charge transfer. Under the friction condition, water molecules can electronically hybridize with surface groups. For example, water acts as an electron acceptor on hydroxylated surfaces and as a donor on carboxylated surfaces, thereby modulating interfacial charge transfer pathways [124, 125]. These findings highlight the crucial role of electron transfer in understanding friction at solid–liquid interfaces.

#### Electron Transfer Mechanisms at Liquid–Solid Interfaces

Traditionally, electron transfer at solid–liquid interfaces has been attributed to differences in the work functions of the contacting materials, a framework that is primarily applicable to metals and semiconductors with well-defined Fermi levels [126–128]. The driving force is governed by the potential difference between solid and liquid ($V_{C} = \frac{{\varphi_{B} - \varphi_{A} }}{e}$), where $\varphi_{A}$ and $\varphi_{B}$ are the work functions of the two substances [129]. In contrast, Wang and his co-workers proposed an electron cloud overlap model (Fig. 8b), which has been recognized as one accepted mechanism for solid–liquid electron transfer [130–132]. In this model, electrons can cross atomic orbital barriers through quantum tunneling, allowing movement and rearrangement of the electron cloud that influences electron transport across the interface. This electron cloud overlap model provides the foundation for investigating friction at solid–water interfaces from a quantum-scale perspective.

Recent studies have revealed the mechanisms of electron transfer at the friction interface between 2D materials and water. Han et al. observed that electron transfer in graphene/MoS_2_ heterostructures significantly increased the slip length by modulating the surface charge redistribution [133]. Xie et al. reported a dual electron transfer during MoS_2_–water contact: initial contact electrification drives electrons into the liquid, while energy released by interfacial bonding can subsequently stimulate electron migration into MoS_2_ (Fig. 8c) [134]. Thus, the direction, magnitude, and dynamic evolution of electron transfer have emerged as key quantum-scale mechanisms for controlling interfacial friction and energy conversion.

Consequently, electron transfer establishes the quantum foundation for charge separation at solid–liquid interfaces, while streaming potentials and capillary flows govern charge redistribution and directional transport. The fluid dynamics and surface chemistry jointly determine long-range charge migration and energy dissipation. Fundamentally, interfacial charge transfer fundamentally influences friction phenomena at the quantum scale. Nevertheless, existing models break down for disordered systems such as amorphous polymers, where electronic orbital disorder invalidates simple transfer. Moreover, multiscale validation is still lacking on whether electron redistribution directly modifies the quantum-correlated friction component and produces a measurable change in macroscopic friction coefficients. Therefore, it is urgently required to develop in situ detection techniques of tracking interfacial evolution in real time and to combine such measurements with simulations to establish cross-scale links from electron transfer events to macroscopic frictional behavior.

Enhancing interfacial charge transfer efficiency is essential for transforming mechanistic insights into practical optimization strategies. Three quantum-scale approaches merit particular consideration:

1) **Material Design Strategy**: Tailoring the electronic structure of either the solid or liquid to optimize the energy level alignment can reduce the electron transfer barrier at the interface. For instance, doping 2D materials can shift the Fermi level, while adjusting the pH of solution can change the redox potential, enhancing the driving force for electron transfer [135, 136].

2) **Interface Engineering Strategy**: Fabricating atomically flat and defect-controlled solid–liquid interfaces can minimize structural disorders that impede charge transport. Techniques such as chemical vapor deposition and in situ interface passivation can create a coherent charge transfer pathway. Experimental results have shown that defect passivation efficiently increases the charge transfer rate [137, 138].

3) **External Field Modulation Strategy**: Utilizing external electric or light field allows dynamical regulation of interfacial charge transfer. A moderate electric field can induce the orientation of polar molecules in the liquid, while light irradiation can excite electrons to higher energy states, promoting non-thermal charge transfer.

### Electron Excitation and Recombination

**Fig. 9:**
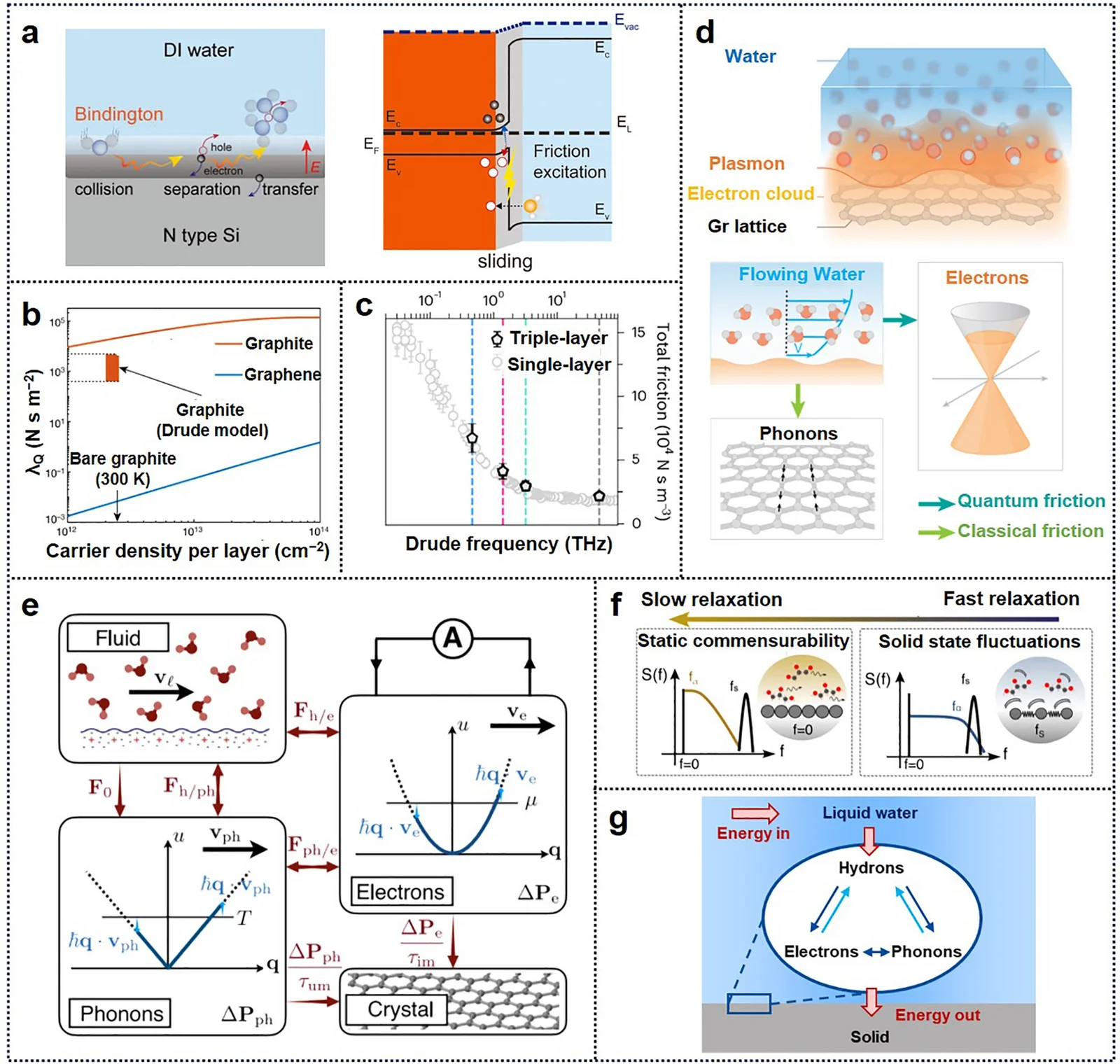
The quantum-scale mechanisms of solid–liquid friction. **a** Solid–liquid friction produces “bindingtons” to excite electron–hole pairs. Reproduced with permission [52, 141]. Copyright © 2021, John Wiley and Sons. Copyright 2021, Elsevier B.V.** b** Electron motion corresponding to a graphite surface plasma and the carrier concentration affects the quantum friction coefficient (*λ*_Q_). Reproduced with permission [49]. Copyright © 2022, Springer Nature. **c** Similarity of total friction of triple-layer and single-layer graphene indicates that friction is essentially determined by the interaction with the first solid layer. Reproduced with permission [143]. Copyright © 2025, Springer Nature.** d** In the classical pathway, energy is transferred to the liquid via phonons; while in the quantum pathway, electrons transfer energy directly to the liquid through Coulomb coupling. Reproduced with permission [79]. Copyright © 2023, Springer Nature. **e** Momentum fluxes in and out of the phonons and electron subsystems need to be balanced in the steady state. Reproduced with permission [146]. Copyright © 2023, American Physical Society. **f** An increased dynamical dissipation occurs when liquid overlaps the solid structure. Reproduced with permission [53]. Copyright © 2024, Springer Nature. **g** Energy dissipation at the liquid–solid interface arises from exchanges between the three species: hydrons, electrons, and phonons. Reproduced with permission [147]. Copyright © 2025, American Institute of Aeronautics and Astronautics

At friction interfaces, mechanical work injected by relative sliding can excite electron–hole pairs and their subsequent recombination, representing an important microscopic channel for frictional energy dissipation [108, 139]. Luo developed the first system for real-time measurement of frictional energy dissipation to resolve the pathways and rates of quantum-scale energy carriers, and found that defects can trap carriers and introduce additional recombination channels, thereby substantially altering frictional behavior [65, 140]. Unfortunately, it remains challenging to extend such a technique directly to solid–liquid interfaces because of stringent experimental requirements, such as low temperature. However, several theoretical and experimental studies have incorporated electron excitation processes as a key mechanism of solid–liquid friction. It has been demonstrated that the energy released during friction at SiO_2_–water interfaces excites electron–hole pairs (Fig. 9a), which are subsequently separated and driven by interfacial electric fields [131, 141]. Lee et al. further demonstrated that ionic layers at solid–liquid interfaces allow more efficient excitation of hot electrons, indicating that electronic excitation constitutes a dissipation channel at friction interfaces [69, 142].

Based on these, quantum friction theory specifically attributed friction to Coulomb interactions arising from resonant coupling between Debye modes in the nonzero-velocity liquid and thermally excited plasmons unique to graphite (Fig. 9b) [49]. Subsequent studies demonstrated that liquid flow can induce another liquid flow behind the wall, a phenomenon known as the “flow tunnel”, whose extent can be tuned via electron excitations in solid (Fig. 9c) [143]. When the frequencies match, the flow tunnel reaches maximum and friction becomes predominantly governed by interactions between the liquid and the topmost solid layer. Consequently, excited electrons can control nanoscale liquid transport by modulating interfacial polarizability, providing a unified physical explanation for ultra-low friction of water on carbon-based surfaces.

The regulatory role of electron excitation in solid–liquid interfacial friction includes not only short-range interactions such as interfacial polarization in water–carbon systems, but also further extends to long-range characteristics involving weak electrostatic forces (van der Waals forces), critically relying on the presence of low-energy, high-momentum electron excitations [144]. Specifically, electron density fluctuations can mediate momentum transfer and increase friction via van der Waals forces even across vacuum gaps, consistent with Levitov’s theory of frictional quantum fluctuations [71, 145]. These non-contact dissipation channels imply that quantum friction modulated by electron excitation may be prevalent at solid–liquid interfaces.

However, several important theoretical issues remain unresolved. First, the competition between chemical-bond reorganization and plasmon closely related to electronic excitation dynamics has not been quantified, leaving the friction energy transfer mechanism unclear. In addition, the role of temperature in tuning resonant coupling conditions lacks a universal description, which limits quantitative predictions of the contribution of electron excitation and recombination to friction.

### Electron–Phonon Coupling

In addition to electrons, phonons are quantum-scale quasiparticles describing the vibrational modes of crystals and are often considered important for the dissipation of mechanical energy during friction [148, 149]. To address the long-standing problem of friction energy dissipation, Chen and his collaborators proposed a phonon dissipation model that provides a clear physical picture of the dynamics of energy dissipation across different friction regimes [150, 151]. Although early studies classified phonon-mediated energy transfer as the classical friction, it has been indicated that phonon-mediated processes often exhibit electronically related signatures. For example, it has been proposed that electron–phonon coupling is a typical channel of friction dissipation and can be probed by ultrafast pump–probe spectroscopy [84, 152].

At solid–liquid interfaces, experiments have demonstrated that phonon-like collective modes of water accelerate the cooling of electrons in graphene via vibration of its hydrogen-bond network, an effect not observed in other polar liquids [79]. This observation highlights an additional contribution of Coulomb interaction arising from electron–phonon coupling to friction, which may be distinct from the “classical friction” (Fig. 9d).

Furthermore, Coquinot and co-workers developed a non-equilibrium perturbative theoretical framework showing that the quantum friction coefficient is determined by both electron excitation and phonon mediation [85, 105, 146]. In this framework, electron–hydron interactions impart momentum to electrons, while electron–phonon scattering facilitates momentum transfer. These processes together control the movement and momentum transfer of electrons in the solid (Fig. 9e). Enhanced electron–phonon scattering can produce a superlinear increase in friction-induced currents, deviating from classical Ohmic scaling. Experimental findings also indicate that solid–liquid friction exhibits a temperature dependence. At low temperatures, friction is dominated by static commensurability (corresponding to a frequency of 0 Hz), whereas at high temperatures, the liquid spectrum overlaps with the phonon peaks of mica, shifting the friction toward fluctuation dominance [53] (Fig. 9f). This directly implicates that solid phonon excitations participate in the energy and momentum exchange at the solid–liquid interface, a process associated with electro-acoustic coupling.

Succi formulated an energy exchange model for solid–liquid systems that quantifies dissipation contributions arising from interactive momentum exchange among hydrons (H), electrons (E), and phonons (P) [147]. Multiscale simulations suggest that quantum-interference effects can reduce friction by more than 50% under certain conditions, providing new avenues for controlling interfacial energy transfer (Fig. 9g). However, experimental implementation remains challenging because of the requirement for synchronous modulation of hydron concentration, carrier density, and phonon mode.

In summary, understanding of solid–liquid friction at the quantum scale is advancing along complementary pathways. Electron transfer and redistribution reconfigure interfacial charge and modulate Coulomb interactions. Electron excitation converts mechanical work into measurable electrical signals, while electron–phonon coupling links electrons and lattice degrees of freedom to mediate momentum and energy exchange. Despite substantial progress, the critical challenge remains to quantitatively characterize the interactions among these mechanisms and establish cross-scale correlations between electron, phonon, and friction coefficients. Addressing these challenges requires coordinated experimental advances, such as in situ ultrafast spectroscopy and operando photoelectron techniques, together with multiscale modeling to build an integrated framework that connects quantum events to macroscopic dissipation.

## Potential Interdisciplinary Applications

The control of solid–liquid friction is the foundation for enhancing energy conversion efficiency. Meanwhile, in-depth exploration of its quantum-scale mechanisms and the establishment of a general theoretical framework for energy conversion between mechanical work and electron motion are critical to uncovering the essence of friction. Based on these mechanisms, the development of effective regulatory strategies and technologies can significantly reduce friction losses at solid–liquid interfaces, thereby facilitating energy-efficient development in areas such as nanofluidic [153, 154], energy technology [155, 156], biomedical systems [157, 158], and tribology [47, 159].

### Nanofluidic Devices

Studies of quantum-scale solid–liquid friction have reframed our understanding of water transport, suggesting that modulation of surface charge or electronic states can be used to tune flow rates. This capability opens routes to nanofluid manipulation and the design of nanoscale valves and pumps, and has driven the development of devices such as nanofluidic transistors, ionic memristors, and quantum-confined sensors.

**Fig. 10:**
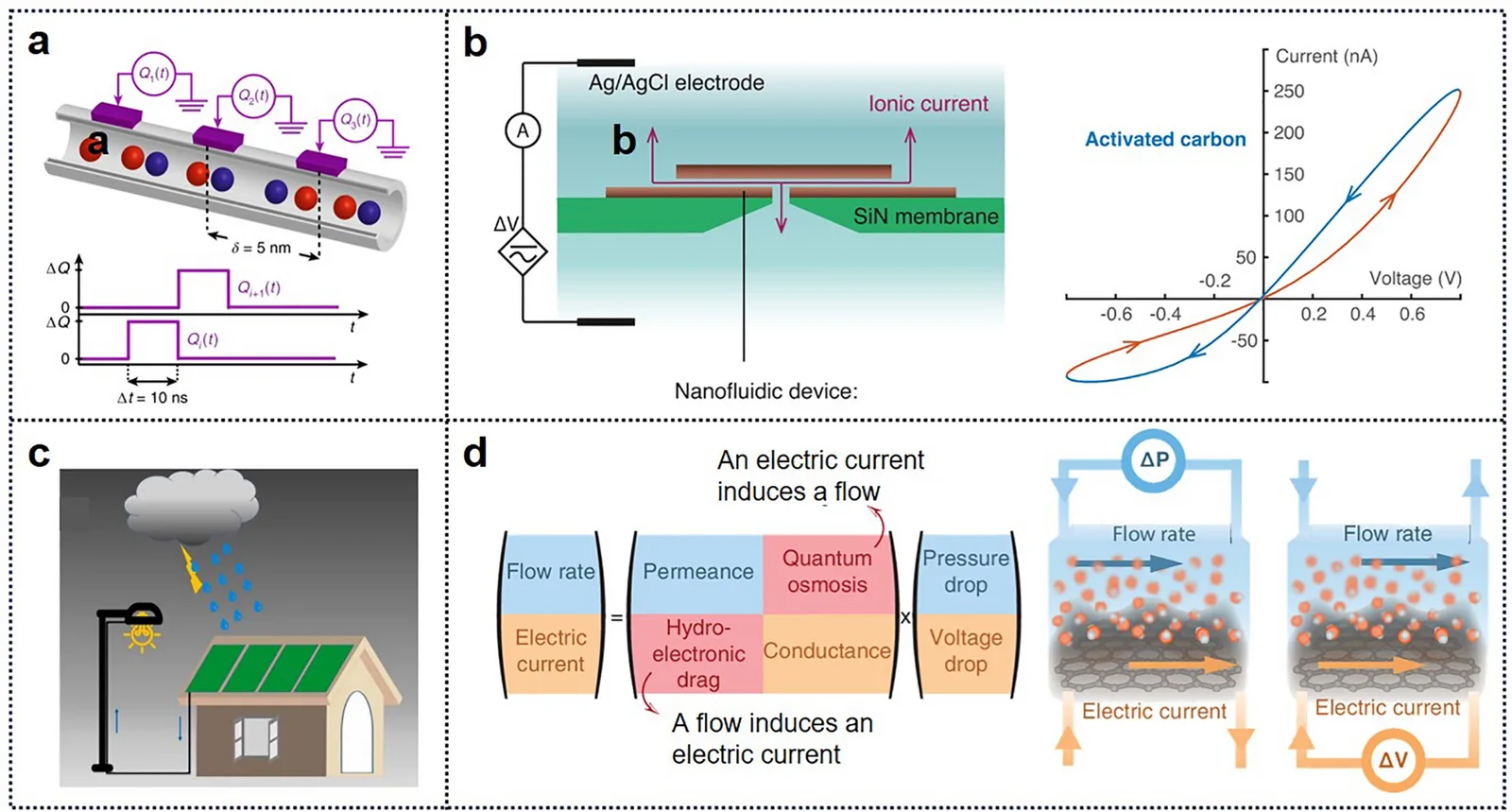
Applications in nanofluids and energy.** a** Oppositely charged ions in gated nanochannels form “Bjerrum pairs”, exploiting the ionic Coulomb blockage formed by their transport to develop ion pumping. Reproduced with permission [161]. Copyright © 2019, Springer Nature. **b** Nanofluidic devices with memory current–voltage characteristics at periodic voltages as a typical example of the memristor effect. Reproduced with permission [165]. Copyright © 2023, American Association for the Advancement of Science.** c** Solid–liquid friction for harvesting raindrop energy. Reproduced with permission [171]. Copyright © 2022, John Wiley and Sons. **d** A pressure drop generates a flow which induces an electric current, thereby inducing a flow through the hydro-electronic friction. Reproduced with permission [105]. Copyright © 2024, PNAS

Recent work demonstrates that voltage-gated nanopores can achieve continuous and reversible control of ion transport by atomically tuning surface charge distributions, a concept termed nanofluidic transistors [160]. This mechanism provides an experimental model for probing how surface electron–ion coupling affects interfacial energy dissipation and consequently affects solid–liquid friction. Building on this principle, ionic pumps have been implemented to achieve single-ion precision transport and filtration (Fig. 10a) [161, 162]. Furthermore, CNTs with extremely low water friction coefficient not only respond to highly localized pressure signals with nanoscale spatial resolution but also exhibit quantum-scale excitation-related nonlinear dependencies of ionic current, suggesting potential applications in highly sensitive localized pressure sensing [163, 164].

In electronics, memristors serve as solid-state analogs of biological synapses because of their tunable conductance and long-term information retention. Nanofluidic memristors based on gating principles (Fig. 10b) lay the groundwork for neuromorphic computing on aqueous electrochemical chips [165]. Related carbon-based microfluidic sensors exploit electron–fluid coupling to enable on-chip detection of minute flows [166]. At Argonne National Laboratory, gate voltages in the sub-volt range have been used to precisely manipulate electronic states in thin-film semiconductor devices [167]. This concept could be extended to liquid or gel media, providing a foundation for low-power semiconductors and quantum devices that exploit solid–liquid interface.

These advances collectively demonstrate that quantum-scale investigations of solid–liquid friction have propelled fluid control beyond theoretical frameworks, gradually advancing the fabrication and application of nanofluidic devices. Nevertheless, two major challenges remain: (1) the difficulty in disentangling and quantifying the relative contributions of quantum effects and classical electrostatic forces to water transport during gating; and (2) interfacial electrochemical degradation under long-term operation, which compromises device performance. Addressing these challenges will require the development of in situ transmission electron microscopy–microfluidics platforms capable of real-time monitoring of coupled multi-physical fields (e.g., flow, electric field). Such strategies will not only enhance nanofluidic device reliability but also inform the design of related energy conversion systems.

### Energy Storage and Conversion

Given the coupling relationship between solid–liquid friction and surface electronic states, interfacial charge has long been exploited for electrokinetic energy conversion. In the context of salinity-gradient energy harvesting, quantum-scale excitations can modulate friction and significantly enhance ion transport efficiency through mechanisms such as surface charge modulation [104, 168, 169]. Nevertheless, current conversion efficiencies remain far from practical targets, primarily due to quantum-scale excitation-induced friction exacerbating performance degradation in high-salinity environments.

Inspired by quantum-scale electro-acoustic effects, Michaelides and Bocquet explored a water hydrodynamic scheme in which electron excitations in graphite layers allow the water on opposite sides of the graphite to flow into one another, producing a “flow tunnel” effect that could be exploited for nanoscale hydropower [143]. This method of harvesting hydrodynamic energy from slow-moving water has been proposed to power ultra-low-power devices [170]. In parallel, electrons excited at the friction interface can convert the mechanical energy of raindrops into measurable currents (Fig. 10c), suggesting large-scale potential for harvesting rain energy [171–173]. Moreover, hydro-voltaic electricity generation driven by water evaporation has been shown to produce continuous power output via spontaneous charge separation at solid–liquid interfaces, offering a viable route for harvesting energy in humid conditions [174].

Recently, Bocquet and Kavokine proposed a physical principle for nanoscale hydropower, illustrated that ion-free flowing liquids can generate electronic currents in solid walls, and on this basis developed a “hydroenergy” to evaluate the efficiency of energy conversion (Fig. 10d) [105]. Analogous to thermoelectric conversion, the efficiency of hydroenergy is governed by a dimensionless figure of merit that combines independently tunable solid and liquid parameters. Lizée further demonstrated very strong ionic–electron coupling at the interface between ionic liquids and single-crystal graphene, implying high interfacial capacitance [175]. However, ionic and electronic transport are typically decoupled over micrometer length scales, and exploiting purely electron transport for power generation remains to be developed.

These findings point to new strategies for blue energy harvesting without electrochemistry and suggest routes to optimize charge transfer in next-generation collectors. Hydrodynamic mechanical energy can be converted to electrical energy by modulating the electronic properties of the solid surface. Despite significant advances, the field faces two major challenges: (1) insufficient experimental validation of quantum-scale energy conversion strategies; (2) theoretical models that do not fully capture energy conversion efficiency and scalability under realistic operating conditions. To further overcome these limitations, integrated approaches combining multiscale simulations and real-time experimental characterization are required to bridge the gap between fundamental mechanisms and practical device implementation.

### Biomedical Equipment

**Fig. 11:**
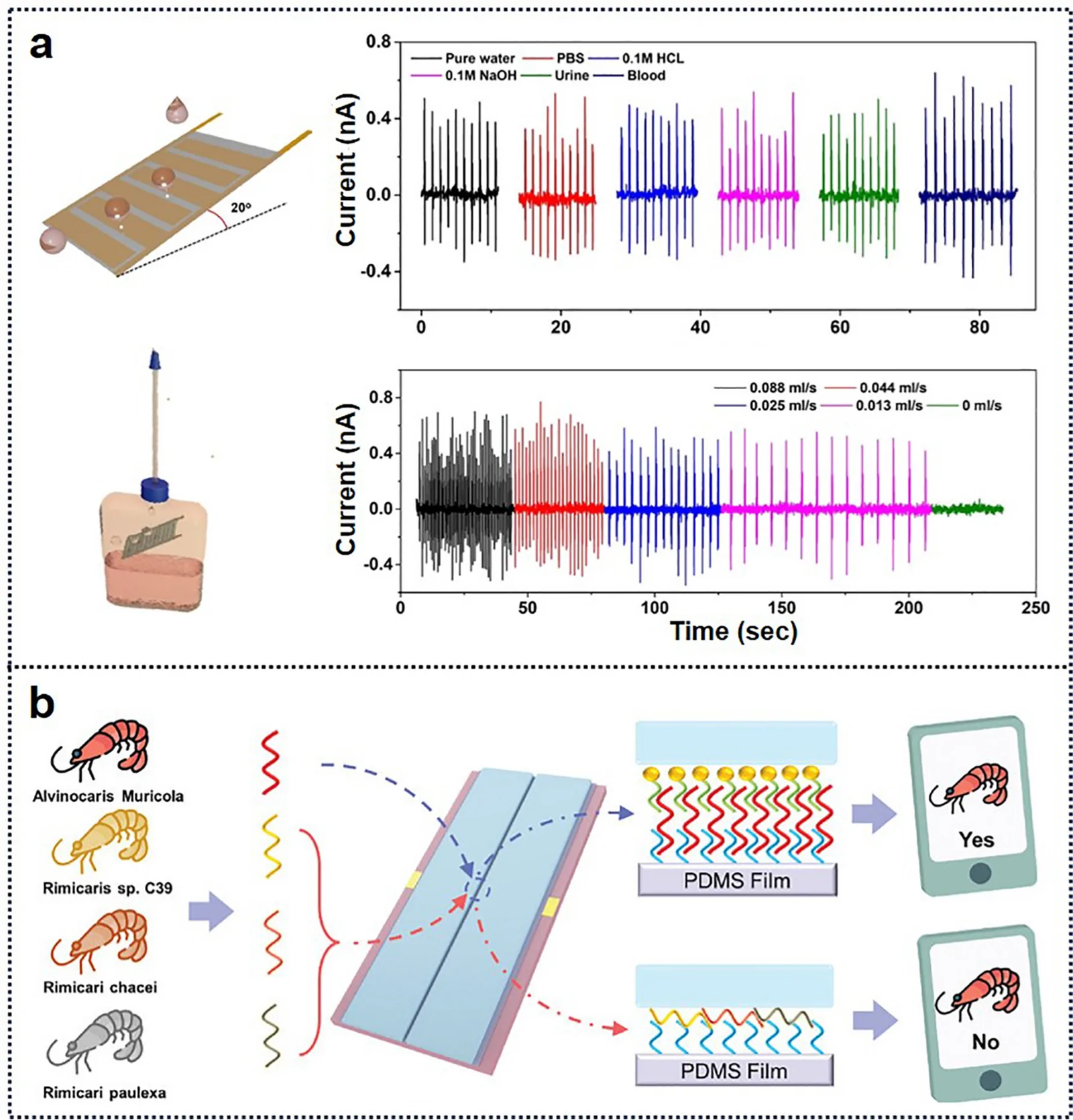
Biomedical applications of quantum-scale solid–liquid friction. **a** Solid–liquid friction developed as self-powered sensors and priming bottle sensors used to monitor the pulse currents generated by drops of blood at different flow rates. Reproduced with permission [178]. Copyright © 2020, American Chemical Society.** b** Liquid–solid friction-based biosensor for DNA barcoding detection of various Alvinocarididae shrimps. Reproduced with permission [179]. Copyright © 2024, John Wiley and Sons

Recent advances in the understanding and control of friction at solid–liquid interfaces on the quantum scale have opened unprecedented application prospects in biomedicine, particularly for highly sensitive biosensors and precision drug delivery systems [176, 177]. Hu et al. investigated the mechanism of electron capture and release at the friction interface of superhydrophobic coatings to develop drainage bottles and prototype smart intravenous infusion monitors for real-time clinical drainage and infusion monitoring (Fig. 11a) [178]. These devices are characterized by simple manufacturing, excellent flexibility, self-cleaning properties, strong adhesion, and high sensitivity. And they display universality for water and various test solutions, including blood. In a different approach, the integration of target DNA barcodes and signal probes with gold nanoparticles (AuNPs) inhibits electron transfer from water to the polydimethylsiloxane (PDMS) tribological interface, substantially reducing the surface charge density and resulting in decreased electric output during friction. Based on this effect, a DNA-barcode biosensor was constructed and employed to identify species such as Alvinocarididae shrimp and Alvinocaris muricola (Fig. 11b) [179].

At the sub-nanometer scale, Kavokine further revealed a distinctive role for carbon nanotubes (CNTs) in flow control: CNTs can spontaneously insert into lipid vesicles. The band gap of the nanotube wall material, rather than pore aperture, was identified as the key determinant of hydrodynamic permeability [70]. This finding highlights the decisive role of electronic structure in the performance of microscopic biological friction interfaces. By precisely tuning osmotic pressure across vesicle membranes, microscopic topology, chemical patterning, and electronic states, extreme fluid-manipulation behaviors such as ultralow friction and unidirectional transport can be realized at bio-interfaces.

Understanding the mechanisms of electron transfer, excitation, and electron–phonon coupling at solid–liquid interfaces provides opportunities for high-sensitivity biomedical devices. However, critical barriers to clinical translation persist: the stability of electron transfer is easily disrupted in complex biological environments, and scalable fabrication of quantum-structured devices remain difficult to achieve. Future progress in this field relies on the development of systems that combine quantum control with biocompatibility, improved device stability, and methodologies to translate quantum-scale solid–liquid friction from fundamental research to clinical applications.

### Super-Lubrication Materials and Surface Engineering

Franzese has argued that elucidating friction at the quantum scale may enable the design of intrinsically low-friction materials [71]. In contrast to conventional lubrication, friction can be markedly reduced without external lubricants by tuning parameters such as electronic structures and atomic arrangement, offering clear environmental benefits.

**Fig. 12:**
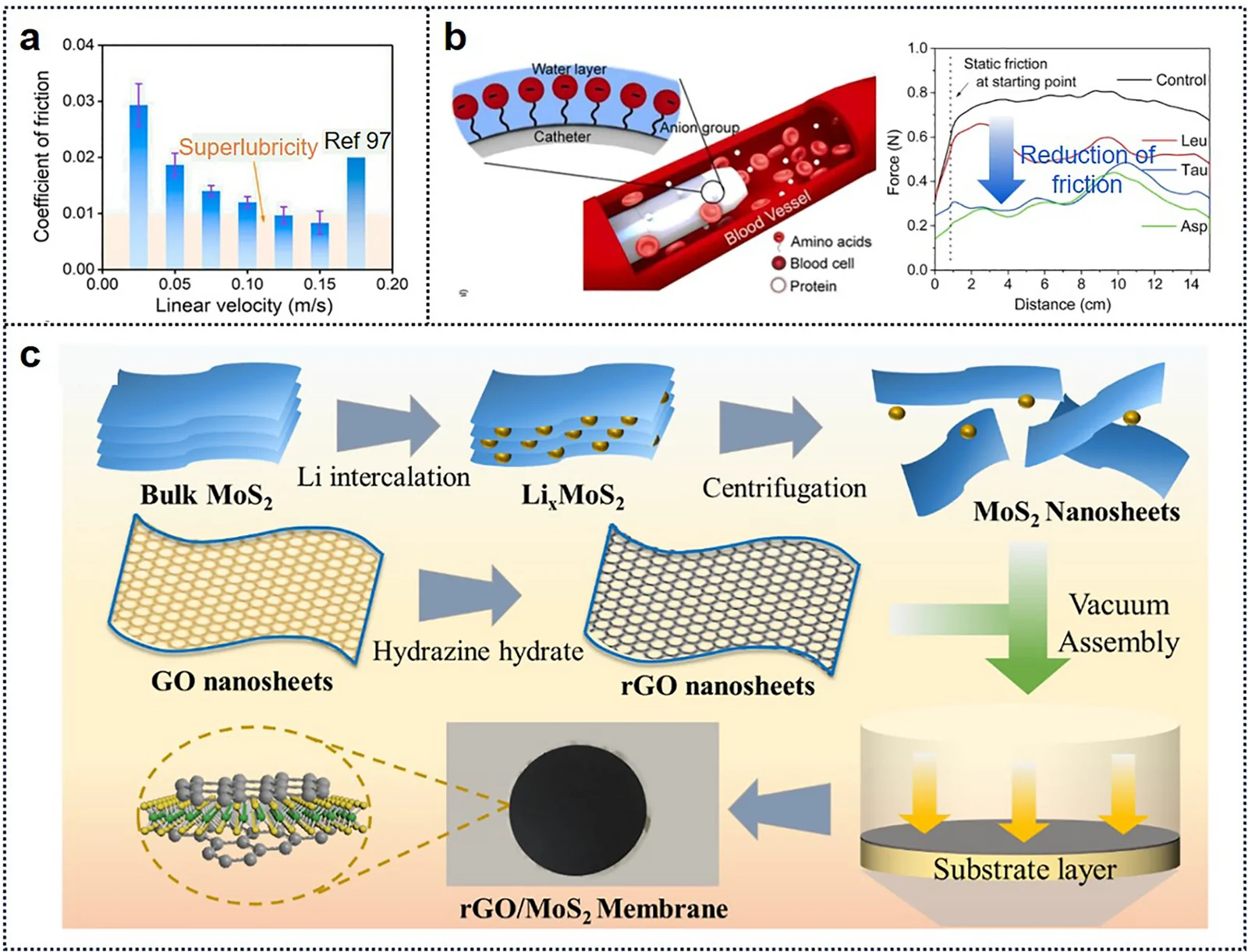
The applications of surface engineering and seawater filtration. **a** Using composite materials has achieved stable superlubricity in seawater at low speeds. Reproduced with permission [186]. Copyright © 2024, Elsevier B.V. **b** Low-friction coating material improves biomedical catheters and effectively reduces the incidence of infection, thrombosis, and tribological damage. Reproduced with permission [187]. Copyright © 2021, Elsevier B.V.** c** Manufacturing process for low-friction rGO/MoS_2_ nanofiltration membranes with improved permeability. Reproduced with permission [184]. Copyright © 2022, American Chemical Society

In solid–liquid systems, research on interfacial friction at the macroscopic scale has already yielded practical applications in super-lubrication materials and biomedical engineering. For example, in marine propulsion systems, super-lubrication interfaces can effectively reduce mechanical friction between bearing components, thereby significantly enhancing operational stability (Fig. 12a). Similarly, in the biomedical field, low-friction materials that optimize the mechanical properties of solid–liquid interfaces can minimize damage to soft tissues and implants while improving the biocompatibility of medical devices (Fig. 12b). However, such superlubricity performance mainly relies on macroscopic approaches, such as mechanical polishing and chemical modification, which are difficult to implement precisely at the nanoscale and often compromise the intrinsic structure of the material.

In contrast, quantum effects enable reversible, non-contact modulation of tribological performance, providing a novel theoretical approach for the targeted development of super-lubrication materials. For instance, water-induced resonance enhancement in graphene, or electron transfer-induced rearrangements of hydration structures and hydrogen-bond networks, can significantly reduce solid–liquid friction [121, 180]. At other 2D material interfaces, defect-enhanced electron–phonon coupling has also been recognized as an effective pathway for regulating friction energy dissipation [152].

Overall, studies of quantum-scale solid–liquid friction establish a foundation for the design of super-lubrication materials and for addressing tribological challenges in engineering and medicine. Nonetheless, several key challenges remain: (1) scalable fabrication of quantum-engineered interfaces is still limited; (2) the long-term stability of quantum effects under friction conditions is not yet fully explored and optimized; (3) atomic-level control over interface structure and functionalization remains challenging. Addressing these issues will require deeper mechanistic insights and the development of a reproducible atomic-scale fabrication technique to achieve stable and scalable super-lubrication materials [181].

### Seawater Desalination and Water Purification

Treating solid–liquid interfacial properties as a many-body problem at the quantum scale is expected to impact filtration and separation of fluid mixtures [33, 49, 182]. First-principles calculations have indicated that charge delocalization and dispersion cause differences in electron cloud interactions, resulting in water molecules passing through ultra-narrow CNTs at rates several orders of magnitude higher than those of salt ions [183]. This phenomenon could reduce the cost and improve the performance of purification filters.

Current strategies focus on optimizing the chemistry and microstructure of nanofiltration membranes to harness interfacial tribology for improved the efficiency of desalination and purification. For example, reduced graphene oxide (rGO)/MoS_2_ membranes prepared via MoS_2_-assisted redox chemistry exhibit expanded nanochannels (Fig. 12c) [184]. These nanochannels induce quantum confinement of interfacial electrons and form weak van der Waals interactions with water molecules to minimize friction while amplifying velocity gradients in water and achieving high flux along with strong salt rejection. Similarly, Ong and co-workers constructed a rough nanoscale selective layer using basic particles of fish scales and elucidated that variations in interfacial electron density modulated permeation flow friction, thereby enhancing solute retention [185].

As the quantum origins of friction become increasingly understood, atomically guided design rules for nanofiltration are emerging. The key challenges include: (1) optimizing membrane materials based on quantum-scale mechanisms to simultaneously achieve high flux, selectivity, and durability; (2) validating these technologies at scales beyond laboratory conditions. Future efforts should focus on atomically informed design principles, enhancement of membrane material performance, and applied validation to enable quantum-scale tribology advances to address global water scarcity effectively.

## Summary and Prospects

In summary, understanding of solid–liquid friction has been extended to the quantum scale, especially with the Coulomb interactions driven by quantum fluctuations. These interactions couple liquid dipole fluctuations to solid electron excitations, which is defined as quantum friction. This concept is not only a microscopic extension of classical tribology but can dominate friction in some systems, such as carbon nanotubes with a small radius. Consequently, the field has shifted from macroscopic models toward a quantum-mechanics-based microscopic paradigm.

Significant progress in solid–liquid friction at the quantum scale has been achieved. In simulation and computation, combinations of multiscale molecular dynamics and first-principles calculations have enabled systematic exploration of quantum effects in friction. Experimentally, terahertz spectroscopy, atomic force microscopy, and nanofluidic transport techniques provide multidimensional characterization of interfacial dynamics. Mechanistically, three interrelated processes form the core of quantum-scale mechanisms governing solid–liquid friction: (i) electron transfer and redistribution, (ii) electron excitation and recombination, and (iii) electron–phonon coupling. These processes show complex competitive or synergistic relationships in different solid–liquid systems, revealing the quantum-scale origins of solid–liquid interface friction and providing a theoretical foundation for interdisciplinary applications in related technologies. In terms of applications, the interdisciplinary and multiscale character of quantum-scale friction positions it as a potential enabler for transformative technologies, including low-power nanofluidic devices, efficient energy storage systems, smart drug delivery platforms, and super-lubrication coatings. Realizing these applications will demand integrated efforts across physics, chemistry, materials science, and engineering, and the establishment of iterative feedback loops that connect fundamental theory with device development.

It is worth emphasizing that current research on quantum-scale friction at solid–liquid interfaces is largely concentrated on 2D material–water systems. This focus stems from the atomically smooth surfaces and unique electronic structures of 2D materials, which strongly enhance quantum effects such as electron–phonon coupling and charge fluctuations in the presence of water. These characteristics make them suitable for both theoretical validation and experimental probing of quantum-scale friction. However, this concentration also reveals a fundamental limitation: the existing frameworks lack universality when extended to more complex solid–liquid systems.

Accordingly, future research should broaden the exploration of quantum-scale friction across diverse solid–liquid systems. Specifically: (i) investigate the manifestations and mechanisms of quantum effects in systems involving materials of different dimensionalities and various liquid media (polar/nonpolar, electrolyte/nonelectrolyte); (ii) develop quantitative quantum-scale friction models that are applicable across multiple interfaces, establishing universal correlations between interfacial quantum-scale properties and friction regulation.

On this basis, two bottlenecks remain in the research and application of quantum-scale friction at solid–liquid interfaces: (1) deviations still exist between simulated interfaces and real interfaces under multi-physics coupling; (2) mature methods for synchronous measurement of multiple physical fields at sub-nanometer spatial and femtosecond temporal resolution are lacking. These dual barriers constrain further progress in the field. Overcoming these barriers requires a focus on the following:

1. In simulation and computation, advancing multiscale hybrid algorithms and improving many-body perturbation frameworks is essential to bridge the gap between quantum precision and engineering complexity, thereby generating experimentally testable predictions.

2. Experimentally, the development of ultra-high spatiotemporal platforms that integrate THz imaging, photoelectron spectroscopy, and integrated nanofluidic/AFM probes will be essential to achieve synchronous measurements and active control of electron dynamics, fluid behavior, and friction.

3. Following improvements in simulation and measurement techniques, bidirectional feedback between them should be fostered to refine understanding of interfacial quantum behavior and friction. Future studies could employ large-scale data analytics to handle datasets produced by ultra-high spatiotemporal measurements, thereby extracting intrinsic connections among quantum-scale excitation processes, mesoscopic fluid flow, and macroscopic friction. This can provide theoretical guidance for reconciling discrepancies between simulations and real observations, thereby addressing measurement limitations.

4. Based on these breakthroughs and incorporating practical applications in key domains such as fluid transport, energy conversion, and super-lubrication materials, innovative technologies and devices based on quantum-scale friction mechanisms will be developed to drive transformative advancements in relevant fields.

The path to fully unravel and utilize solid–liquid friction continues, with each new discovery opening avenues to address key challenges in energy, environment, and health.
